# Molecular identification of the key starch branching enzyme-encoding gene *SBE2.3* and its interacting transcription factors in banana fruits

**DOI:** 10.1038/s41438-020-0325-1

**Published:** 2020-07-01

**Authors:** Hongxia Miao, Peiguang Sun, Qing Liu, Juhua Liu, Caihong Jia, Dongfang Zhao, Biyu Xu, Zhiqiang Jin

**Affiliations:** 1 Key Laboratory of Tropical Crop Biotechnology, Ministry of Agriculture, Institute of Tropical Bioscience and Biotechnology, Chinese Academy of Tropical Agricultural Sciences, 571101 Haikou, People’s Republic of China; 2 Key Laboratory of Genetic Improvement of Bananas, Haikou Experimental Station, Chinese Academy of Tropical Agricultural Sciences, 571101 Haikou, Hainan Province People’s Republic of China; 3 Commonwealth Scientific and Industrial Research Organization Agriculture and Food, Canberra, ACT 2601 Australia; 4 College of Horticulture, Nanjing Agricultural University, 210095 Nanjing, People’s Republic of China

**Keywords:** Plant molecular biology, Transcriptomics

## Abstract

Starch branching enzyme (SBE) has rarely been studied in common starchy banana fruits. For the first time, we report here the molecular characterization of seven *SBE* (*MaSBE*) and six *SBE* (*MbSBE*) genes in the banana A- and B-genomes, respectively, which could be classified into three distinct subfamilies according to genome-wide identification. Systematic transcriptomic analysis revealed that six *MaSBE*s and six *MbSBE*s were expressed in the developing banana fruits of two different genotypes, BaXi Jiao (BX, AAA) and Fen Jiao (FJ, AAB), among which *MaSBE2.3* and *MbSBE2.3* were highly expressed. Transient silencing of *MaSBE2.3* expression in banana fruit discs led to a significant decrease in its transcription, which coincides with significant reductions in total starch and amylopectin contents compared to those of empty vector controls. The suggested functional role of *MaSBE2.3* in banana fruit development was corroborated by its transient overexpression in banana fruit discs, which led to significant enhancements in total starch and amylopectin contents. A number of transcription factors, including three auxin response factors (ARF2/12/24) and two MYBs (MYB3/308), that interact with the *MaSBE2.3* promoter were identified by yeast one-hybrid library assays. Among these ARFs and MYBs, MaARF2/MaMYB308 and MaARF12/MaARF24/MaMYB3 were demonstrated via a luciferase reporter system to upregulate and downregulate the expression of *MaSBE2.3*, respectively.

## Introduction

Plant starch, which consists of two glucan polymers, linear amylose and branched amylopectin, is a major supplier of human nutrition and calories. Starch is commonly found in cereal grains and potato tubers^1,2^ as well as in some fresh fruits, such as mango (*Mangifera indica*)^3^, kiwifruit (*Actinidia deliciosa*)^4^, and banana (*Musa acuminata*)^5–7^. As the main component of the total starch in the storage organs of the abovementioned plant species, amylopectin is primarily catalyzed by soluble starch synthase, debranching enzyme, and starch branching enzyme (SBE)^8^. Of these three classes of major enzymes, SBE mainly controls the branching pattern of amylopectin and influences the amylopectin yield and, ultimately, the nutritional value of those plants^9–11^.

Variable numbers of *SBE*s have been isolated in several higher plant species based on genomic data: three have been identified in maize (*Zea mays*), rice (*Oryza sativa*), sorghum (*Sorghum bicolor*), barley (*Hordeum vulgare*), and potato (*Solanum tuberosum*); two have been identified in Arabidopsis (*Arabidopsis thaliana*); six in cassava (*Manihot esculenta*); and seven in wheat (*Triticum aestivum*)^12–15^. Furthermore, *SBE*s can be generally classified into no less than three groups—SBEI, II, and III, each of which typically contains a C-terminal all-beta domain and an alpha-amylase domain^16^. SBEI condenses amylose to long glucan chains^13^, while SBEII is mainly associated with the biosynthesis of amylopectin using short glucan chains as a substrate^13,17^. However, the functional role of SBEIII remains ambiguous despite reports of its increased activity in the process of wheat grain development^18^.

A number of expression and functional studies have further revealed the multifunctional aspect of *SBE*s in both amylopectin biosynthesis and plant development. For instance, the temporal expression of *SBE1* was found to be responsible for amylopectin accumulation in apple fruits and in maize seedling development^12,19^. *SBEII* expression is involved in nut development in chestnut (*Castanea mollissima*)^20^ and seed development in rice^21^. A study on the kidney bean (*Phaseolus vulgaris*) mutant *pvsbe2* suggested that SBEII activity is important for both amylopectin biosynthesis and maturation during the middle and late stages of seed development^22^. Further, the expression of *OsSBEIIa* was found to regulate the sugary endosperm trait during development and maturity in the *Sugary Endosperm* rice mutant^23^. In maize, *SBEIIa*-defective mutants exhibit a leaf-senescence phenotype as the result of the reduction in the proportion of branched starch^24^. Taken together, all of these studies imply that the expression of *SBE* is a critical factor in amylopectin biosynthesis and plant growth and development.

A number of transcription factors (TFs) regulating *SBE* expression have been identified. In maize, the expression of numerous *SBE* genes was upregulated when *ZmNAC36* was transiently overexpressed^25^, while the protein levels of maize *SBE*s were significantly reduced in the endosperm-specific TF *opaque2* mutant and prolamin-box-binding factor (*PBF*) mutant^26^. In rice, OsbZIP58 was found to upregulate *SBE1* expression through its interaction with the *SBE1* promoter^27^. Taken together, all of these studies indicated that *SBE* expression is regulated by the coordination of multiple TFs.

Banana is a popular fresh starchy fruit worldwide and constitutes the principal staple food in several African countries^28^. Amylopectin accounts for approximately 70–80% of the total starch content on a dry weight basis in unripe banana fruits^6,7,29^. The biosynthesis and accumulation of amylopectin in banana fruit is of crucial importance in terms of fruit quality, yield, and nutritional value. Genome-wide identification of key candidate genes associated with amylopectin biosynthesis is therefore necessary to enhance the yield and improve the quality of banana fruits^6,30,31^. In the current study, we report the identification of seven and six distinct *SBE* members from the banana A- and B-genomes, respectively, which are henceforth referred to as *MaSBE* and *MbSBE*. The molecular characteristics, phylogenetic relationships, conserved domains, gene structures, and spatiotemporal expression patterns were investigated in the two different banana genotypes. The potential functionality of *MaSBE2.3* was studied through both transient virus-induced gene silencing (VIGS) and overexpression in banana fruit discs. Three auxin response factors (ARF2/12/24) and two MYBs (MYB3/308) that regulate *MaSBE2.3* expression were identified using the yeast one-hybrid (Y1H) method and luciferase (LUC) activity analysis. Together, these studies may enhance our understanding of the members of the *SBE* gene family involved in amylopectin biosynthesis and fruit development and pave the way for the genetic improvement of the quality, yield, and nutritional traits of banana.

## Materials and methods

### Plant material

The two banana genotypes used in this experiment were Fen Jiao (FJ, *M. acuminata*, AAB, cultivar Fenjiao) and BaXi Jiao (BX, *M. acuminata*, AAA, cultivar Cavendish), which were planted at the Danzhou Banana Germplasm Centre (Hainan, China, 19 N, 109 E). BX fruits are characterized by their high yield, superior quality, and long shelf-life, whereas FJ fruits are characterized by their good flavor, rapid ripening, and strong disease resistance. According to the published classification of banana fruit developmental stages by Ram et al.^32^, we selected three representative stages corresponding to pulp initiation at 0 day after inflorescence emergence from the pseudostem (DAF), pulp growth (20 DAF), and maturation (80 DAF) for *SBE* temporal expression analysis. Following harvest at 80 DAF, BX fruits were stored for 0, 8, and 14 days post-harvest (DPH), while FJ fruits were stored for 0, 3, and 6 DPH prior to expression analysis of *SBE* during fruit ripening. These fruits can also be distinguished by their color: peel green, peel yellowish green, and peel yellow. A spatial gene expression analysis of *SBE*s was also performed on select banana tissues, including root, leaf, and fruit tissues, at 80 DAF. These expression studies were conducted for three replicates.

### Genome-wide investigation and phylogenetic tree construction of SBE family proteins

SBE amino acid sequences were obtained from the *M. acuminata* (DH-Pahang, AA genotype, 2*n* = 22) database^5^ and *Musa balbisiana* (DH-PKW, BB genotype, 2*n* = 22) database^7^. The putative amino acid sequences of SBE from *A. thaliana*, *T. aestivum*, *Z. mays*, *O. sativa*, *Solanum lycopersicum*, *S. tuberosum*, and *Vitis vinifera* were retrieved from The Arabidopsis Information Resource, the wheat genome, the Maize Genetics and Genomics database, the Rice Genome Annotation Project, the Sol Genomics Network, the Potato Genome Sequence Consortium, and the grapevine genome, respectively. All bioinformatic analytical databases and websites used are listed in Table S1. The banana SBEs, together with SBEs from the *A. thaliana*, *T. aestivum*, *Z. mays*, *O. sativa*, *S. tuberosum*, *S. lycopersicum*, and *V. vinifera* searches, were analyzed using the BLAST software. The accession numbers of the SBEs from the abovementioned plant species are presented in Table S2. The chromosome distribution of MaSBEs and MbSBEs was analyzed by the Circos software. The SBE protein sequences derived from these diverse plant species were used to construct a phylogenetic tree using the ClustalX 2.0 tool (http://downloads.fyxm.net/Clustal-x-58923.html) and MEGA 5.0 software based on 1000 bootstrap replicates^31^.

### Characterization of conserved domains and exon–intron structure

The isoelectric point and molecular mass of the *SBE*s were calculated using ExPASy, and the data are presented in Table S3. The conserved domains and SBE proteins were predicted and annotated by using the NCBI database and MEME software. The polypeptides were deduced and analyzed by PeptideMass and BLASTP software (Table S4). The genomic structure of the *SBE* genes was deduced by using the Gene Structure Display Server. The *MaSBE* promoter was obtained from the banana A-genome database within the Banana Genome Hub. The predicted transcription start sites and *cis*-acting elements were identified by searching the Berkeley Drosophila Genome Project database and the PlantCARE website, respectively.

### RNA deep sequencing and transcriptomic analysis

Various banana plant organs, including the roots, leaves, and fruits, at 80 DAF in both BX and FJ genotypes were collected for spatial transcriptomic analysis. The pulp of banana fruits at various developmental and ripening stages, including that of BX and FJ at 0, 20, and 80 DAF, that of BX at 8 and 14 DPH, and that of FJ at 3 and 6 DPH, were collected for transcriptome sequencing at different time points.

RNA-sequencing (RNA-seq) libraries were constructed using total RNA samples prepared by the use of a plant RNAout Kit (TIANGEN Biotech, Beijing, China). Deep sequencing was performed by using the GAII platform (Illumina, Inc., San Diego, CA) according to the protocol provided by the manufacturer. The adapter sequences and low-quality sequences were removed by using the FASTX toolkit and FastQC, respectively. After mapping the clean reads to both the A- and B-genome banana databases, transcriptome assemblies were constructed by using Cufflinks. The gene expression level was assessed as reads per kilobase pair of transcripts per million reads (RPKM). The DESeq package was applied to screen differentially expressed genes. There were two technical replicates and three biological replicates.

### Quantitative real-time polymerase chain reaction (qRT-PCR) analysis

The spatiotemporal expression patterns of banana *SBE*s revealed by transcriptome sequencing were further identified through qRT-PCR using an Mx3000P system (Stratagene, San Diego, CA). The primer pair quality was verified according to melting curve analysis, with amplification efficiencies ranging from 0.9 to 1.1, and agarose gel electrophoresis (Table S5). *Actin* (GenBank accession No. EF672732) and *UBQ2* (GenBank accession No. HQ853254) were used as the internal reference genes. The relative gene expression level was calculated according to the 2^−ΔΔ*C*T^ method^33^. There were three replicates included.

### Vector construction and VIGS in banana fruit discs

For VIGS vector construction, the entire coding region of *MaSBE2.3* (2502 bp in length) was amplified by PCR and verified by DNA sequencing (the primers used are listed in Table S6; the sequence is listed in Fig. S1) prior to insertion between the *Eco*R I and *Xho* I restriction enzyme sites in pTRV2^34^, yielding pTRV2-MaSBE2.3, which was subsequently introduced into Agrobacterium (*Agrobacterium tumefaciens* strain GV3101) together with pRTV1. Surface-sterilized BX banana fruit (80 DAF) discs that were 1–2 mm thick were then vacuum infiltrated with the Agrobacterium solution (OD_600_ = 0.6) prior to cocultivation on Murashige and Skoog (MS) media at 30 °C for 0, 1, 2, or 3 days^35^, followed by iodine–potassium iodide (I_2_-KI) staining^36^. In addition to I_2_-KI staining, assessment of the transcript levels of the downregulated genes and the measurements of total starch and amylopectin contents in these agroinfiltrated banana fruit discs were also carried out. Biologically independent replicates were assessed in triplicate.

### Vector construction and transient overexpression in banana fruit discs

For the transient overexpression vector, the entire coding region of *MaSBE2.3* was inserted into a pCAMBIA1304 vector (Cambia, Canberra, Australia) behind the CaMV 35S promoter in the sense orientation using the restriction enzymes *Bgl* I and *Spe* I. Following confirmation by DNA sequencing, the recombinant pCAMBIA1304-MaSBE2.3 plasmid was transformed into *A*. *tumefaciens* GV3101 cells. The cells of the *A*. *tumefaciens* strain were diluted to an OD_600_ of 0.60 in infiltration buffer (200 mM acetosyringone, 10 mM 2-(*N*-Morpholino)ethanesulfonic acid, 10 mM MgCl_2_), in which the BX banana fruit discs (80 DAF) were soaked^34^. Following a period of culture on MS media at 30 °C for 3 days, the agroinfiltrated banana fruit discs were subjected to I_2_-KI staining, transcription analysis, and total starch and amylopectin content evaluations. The biologically independent transformations were performed in triplicate.

### Measurement of total starch content

The banana pulp was soaked for 10 min in 1.5% sodium bisulfite solution to prevent browning prior to drying for 24 h at 40 °C^37^, after which the material was ground into powder. One hundred milligrams of powder was washed in 5 mL of 80% ethanol and centrifuged for 5 min at 4000 rpm. Following the removal of the supernatant, the pellet was washed in 5 mL of 80% Ca(NO_3_)_2_ twice and then centrifuged for 5 min at 4000 rpm. The total starch content was calculated as the percentage per gram of dry sample, which was calculated according to a 100 μg/mL starch standard solution (Sigma, S-2630) with an absorbance of 620 nm^38^. Three replicates were measured.

### Measurement of amylopectin content

In total, 9 mL of 1 M sodium hydroxide and 1 mL of 95% ethanol were added to a sample of 100 mg of banana powder, after which the mixture was incubated at 40 °C for a period of 24 h. Afterward, 5 mL of the mixture, 2 mL of 0.2 N I_2_-KI solution, and 1 mL of 1 M acetic acid were mixed together prior to further dilution to 100 mL with distilled water in a new volumetric flask. The amylopectin content was calculated as the percentage per gram of dry sample based on a 1 mg/mL amylopectin standard solution (Sigma-Aldrich, catalog number 10120) measured at 533 nm. The measurement was carried out for three replicates.

### cDNA library construction and Y1H library screening

Total RNA was extracted from the BX banana pulp samples at 0, 20, and 80 DAF by a plant RNAout Kit (TIANGEN Biotech) and then applied to cDNA library construction by using a Mathchmaker Gold Yeast One-Hybrid Library Screening System Kit (Clontech, Mountain View, CA). The putative *MaSBE2.3* promoter, which consists of a genomic DNA fragment 1824 bp in length, immediately upstream of the *MaSBE2.3* open reading frame (ORF; the primers used are listed in Table S6; the sequence is listed in Fig. S2) was inserted into a pAbAi vector (Clontech) using the restriction enzymes *Sac* I and *Xho* I. The bait and pGADT7-Rec prey vectors together with the cDNA library were cotransformed into the Y1HGold (Clontech) cells, which were subsequently cultured on SD/-Leu+AbA^200^ media for 3 days at 30 °C. Selected positive colonies were further verified via PCR and sequence analyses. The identified TF genes were annotated in the banana A-genome database^5^.

### Y1H assays

To identify the individual interaction between the *MaSBE2.3* promoter and a single TF, the *MaSBE2.3* promoter was inserted into a pAbAi vector to form a bait construct (Clontech). A prey construct was built by inserting the ORFs of candidate TF genes (the primer sequences used are listed in Table S6; the sequences are listed in Fig. S3) into a pGADT7 AD vector. Both the prey and bait gene constructs were cotransformed into cells of the Y1HGold yeast strain (Clontech), which were subsequently grown on SD/-Leu+AbA^200^ selective media at 30 °C for 3 days.

### Analysis of dual LUC activity

The *MaSBE2.3* promoter was cloned into a pGreenII 0800-REN-LUC vector as described by Hellens et al.^39^. The ORFs of MaARF2/12/24 and MaMYB3/308 were each inserted into a pGreenII 62Sk vector separately, which were subsequently transformed into the *A. tumefaciens* GV3101 strain. *A. tumefaciens* cultures harboring the pGreen-MaSBE2.3 promoter vector and *A. tumefaciens* cultures containing pGreenII62Sk-MaARFs or pGreenII62Sk-MaMYBs were mixed together at a ratio of 1:7 (v/v) prior to cocultivation with banana fruit discs by soaking and shaking. The luciferase and REN-LUC activities following cocultivation for a period of 3 days were analyzed by using a dual-LUC reporter assay system (Promega, Madison, WI). Three replicates were measured.

### Statistical analysis methods

SPSS 10.0 software (SPSS Inc., Chicago, IL) was used for the statistical analyses. The least significant difference test was used for one-way analysis of variance (ANOVA). Student’s *t* tests were applied to test the differences between the means revealed by ANOVA. Each sample consisted of three replicates, and *p* < 0.05 and *p* < 0.01 were recognized as statistically significant and highly significant, respectively.

## Results

### Genome-wide characterization and phylogenetic analysis of banana *SBE* genes

**Fig. 1:**
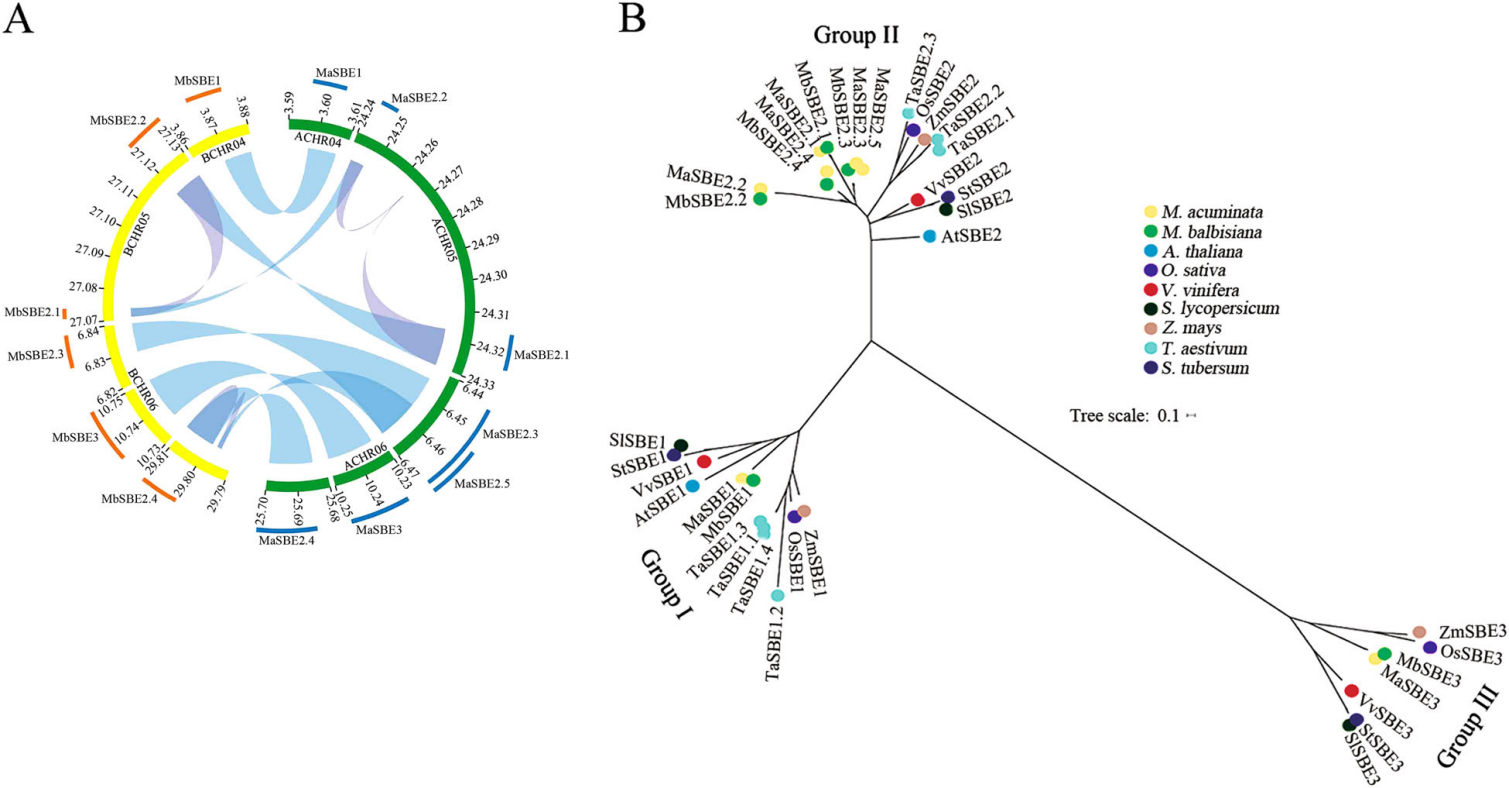
Genome-wide identification and phylogenetic tree of banana *SBE*s and their orthologs from Arabidopsis, rice, grape, tomato, maize, wheat, and potato. **a** Circos image generated by the Circos software. **b** Phylogenetic tree constructed by MEGA 5.0. The *SBE*s were divided into three subfamilies: groups I, II, and III. ACHR04/05/06 represent chromosomes 4, 5, and 6 from the banana A-genome, respectively, and BCHR04/05/06 represent chromosomes 4, 5, and 6 from the banana B-genome, respectively. The yellow, green, blue, purple, red, black, orange, light blue, and dark purple circles represent *SBE*s from *Musa acuminata*, *Musa balbisiana*, *Arabidopsis thaliana*, *Oryza sativa*, *Vitis vinifera*, *Solanum lycopersicum*, *Zea mays*, *Triticum aestivum*, and *Solanum tuberosum*, respectively

Seven *M. acuminata* MaSBE (MaSBE-1, -2.1, -2.2, -2.3, -2.4, -2.5, and -3) and six *M. balbisiana* MbSBE (MbSBE-1, -2.1, -2.2, -2.3, -2.4, and -3) amino acid sequences were obtained from the banana A- and B-genome databases^5,7^, respectively. Among the 11 chromosomes, MaSBE1/MbSBE1, MaSBE2.1/2.2/MbSBE2.1/2.2, and MaSBE2.3/2.4/2.5/3/MbSBE2.3/2.4/3 were distributed on chromosomes 4, 5, and 6, respectively (Fig. 1a). The number of amino acids in the putative MaSBE and MbSBE proteins varied from 162 (MbSBE2.2) to 924 (MbSBE3), with relative molecular masses ranging from 17.314 (MbSBE2.2) to 106.701 (MbSBE3) kDa and isoelectric points ranging from 4.87 (MbSBE2.1) to 7.74 (MaSBE2.2) (Table S3). The high-level variation of *MaSBE*s and *MbSBE*s in the numbers of amino acids, molecular mass, and isoelectric points may possibly reflect their functional divergence in various biological processes.

To understand the evolution of the SBE proteins in banana, a phylogenetic tree was constructed by comparing all seven MaSEB and six MbSBE amino acid sequences containing GlgB domains (Tables 1 and 2) together with the amino acid sequences of each of the following: three *O. sativa* OsSBEs, *Z. mays* ZmSBEs, *S. tuberosum* StSBEs, *V. vinifera* VvSBEs, and *S. lycopersicum* SlSBEs; two *A. thaliana* AtSBEs; and seven *T. aestivum* TaSBEs (Fig. 1b). *MaSBE*s and *MbSBE*s clustered into three groups (I–III). MaSBE1/MbSBE1 and MaSBE3/MbSBE3 were found in group I and group III, respectively. The majority of banana *SBE*s, including five MaSBE (MaSBE-2.1, -2.2, -2.3, -2.4, and -2.5) and four MbSBE (MbSBE-2.1, -2.2, -2.3, and -2.4) members, were present in group II. It is likely that the banana *SBE*s in group II have undergone significant gene duplication and possible functional diversification throughout the evolutionary process.

**Table 1:** Conserved domains and functional annotation of banana MaSBE proteins

Protein name	Accession name	Description	Interval	*E*-value
MaSBE1	PLN02447	1,4-alpha-glucan-branching enzyme	72–770	0.00E+00
	PF00128	Alpha-amylase domain	239–647	0.00E+00
	GlgB	1,4-alpha-glucan branching enzyme	144–768	7.11E−107
	Branching_enzyme	Alpha-1,4-glucan 6-glycosyltransferase	120–723	2.74E−63
	CBM_48	Carbohydrate-binding module 48 (Isoamylase N-terminal domain)	132–216	1.99E−23
	PF02806	C-terminal all-beta domain	283–433	3.75E−13
MaSBE2.1	PLN02447	1,4-alpha-glucan-branching enzyme	70–768	0.00E+00
	PF00128	Alpha-amylase domain	237–645	0.00E+00
	GlgB	1,4-alpha-glucan branching enzyme	142–766	2.42E−106
	Branching_enzyme	Alpha-1,4-glucan 6-glycosyltransferase	118–721	8.27E−63
	CBM_48	Carbohydrate-binding module 48 (Isoamylase N-terminal domain)	130–214	2.41E−23
	PF02806	C-terminal all-beta domain	281–431	4.95E−13
MaSBE2.2	GlgB	1,4-alpha-glucan branching enzyme	142–215	9.83E−39
	E_set_GBE_euk_N	N-terminal Early set domain	204–215	8.45E−03
MaSBE2.3	PLN02447	1,4-alpha-glucan-branching enzyme	111–830	0.00E+00
	PF00128	Alpha-amylase domain	304–719	0.00E+00
	GlgB	1,4-alpha-glucan branching enzyme	197–830	5.41E−123
	Branching_enzyme	Alpha-1,4-glucan 6-glycosyltransferase	187–827	2.55E−62
	CBM_48	Carbohydrate-binding module 48 (Isoamylase N-terminal domain)	736–831	5.79E−24
	PF02806	C-terminal all-beta domain	348–419	4.53E−15
MaSBE2.4	PLN02447	1,4-alpha-glucan-branching enzyme	123–840	0.00E+00
	PF00128	Alpha-amylase domain	310–725	0.00E+00
	GlgB	1,4-alpha-glucan branching enzyme	203–837	9.07E−124
	Branching_enzyme	Alpha-1,4-glucan 6-glycosyltransferase	201–828	3.19E−63
	CBM_48	Carbohydrate-binding module 48 (Isoamylase N-terminal domain)	743–838	2.66E−24
	PF02806	C-terminal all-beta domain	354–425	2.01E−14
MaSBE2.5	PLN02447	1,4-alpha-glucan-branching enzyme	1–501	0.00E+00
	PF00128	Alpha-amylase domain	1–390	0.00E+00
	GlgB	1,4-alpha-glucan branching enzyme	10–501	1.28E−99
MaSBE3	PLN02960	Alpha-amylase	9–906	0.00E+00
	PF00128	Alpha-amylase domain	388–787	0.00E+00
	GlgB	1,4-alpha-glucan branching enzyme	325–900	1.93E−83
	Branching_enzyme	Alpha-1,4-glucan 6-glycosyltransferase	341–891	1.62E−51
	Alpha-amylase_C	Alpha-amylase_C	806–901	2.55E−17
	PF02806	C-terminal all-beta domain	424–572	1.49E−13

**Table 2:** Conserved domains and functional annotation of banana MbSBE proteins

Protein name	Accession name	Description	Interval	*E*-value
MbSBE1	PLN02447	1,4-alpha-glucan-branching enzyme	77–775	0.00E+00
	PF00128	Alpha-amylase domain	244–652	0.00E+00
	GlgB	1,4-alpha-glucan branching enzyme	149–773	1.34E−107
	Branching_enzyme	Alpha-1,4-glucan 6-glycosyltransferase	125–728	5.06E−63
	CBM_48	Carbohydrate-binding module 48 (Isoamylase N-terminal domain)	137–221	2.00E−23
	PF02806	C-terminal all-beta domain	288–438	2.19E−13
MbSBE2.1	PLN02447	1,4-alpha-glucan-branching enzyme	52–476	0.00E+00
	PF00128	Alpha-amylase domain	51–361	0.00E+00
	GlgB	1,4-alpha-glucan branching enzyme	64–473	7.40E−63
	CBM_48	Carbohydrate-binding module 48 (Isoamylase N-terminal domain)	65–464	8.14E−27
	PF02806	C-terminal all-beta domain	379–474	1.92E−23
MbSBE2.2	GlgB	1,4-alpha-glucan branching enzyme	142–162	9.90E−03
	E_set_GBE_euk_N	N-terminal Early set domain	204–215	8.45E−03
MbSBE2.3	PLN02447	1,4-alpha-glucan-branching enzyme	105–787	0.00E+00
	PF00128	Alpha-amylase domain	310–724	0.00E+00
	GlgB	1,4-alpha-glucan branching enzyme	203–787	2.61E−101
	Branching_enzyme	Alpha-1,4-glucan 6-glycosyltransferase	201–786	8.39E−52
	CBM_48	Carbohydrate-binding module 48 (Isoamylase N-terminal domain)	205–289	8.94E−23
	PF02806	C-terminal all-beta domain	354–425	1.44E−01
MbSBE2.4	PLN02447	1,4-alpha-glucan-branching enzyme	148–899	0.00E+00
	PF00128	Alpha-amylase domain	356–787	0.00E+00
	GlgB	1,4-alpha-glucan branching enzyme	249–899	4.09E−119
	Branching_enzyme	Alpha-1,4-glucan 6-glycosyltransferase	239–896	4.31E−60
	CBM_48	Carbohydrate-binding module 48 (Isoamylase N-terminal domain)	805–900	7.35E−24
	PF02806	C-terminal all-beta domain	400–471	2.60E−15
MbSBE3	PLN02960	Alpha-amylase	21–924	0.00E+00
	PF00128	Alpha-amylase domain	390–755	4.93E−155
	GlgB	1,4-alpha-glucan branching enzyme	325–918	1.45E−61
	Branching_enzyme	Alpha-1,4-glucan 6-glycosyltransferase	341–882	4.04E−32
	Alpha-amylase_C	Alpha-amylase_C	830–919	2.05E−16
	PF02806	C-terminal all-beta domain	426–498	7.63E−13

### Exon–intron structural characteristics and conserved domain annotations of the banana SBE family members

**Fig. 2:**
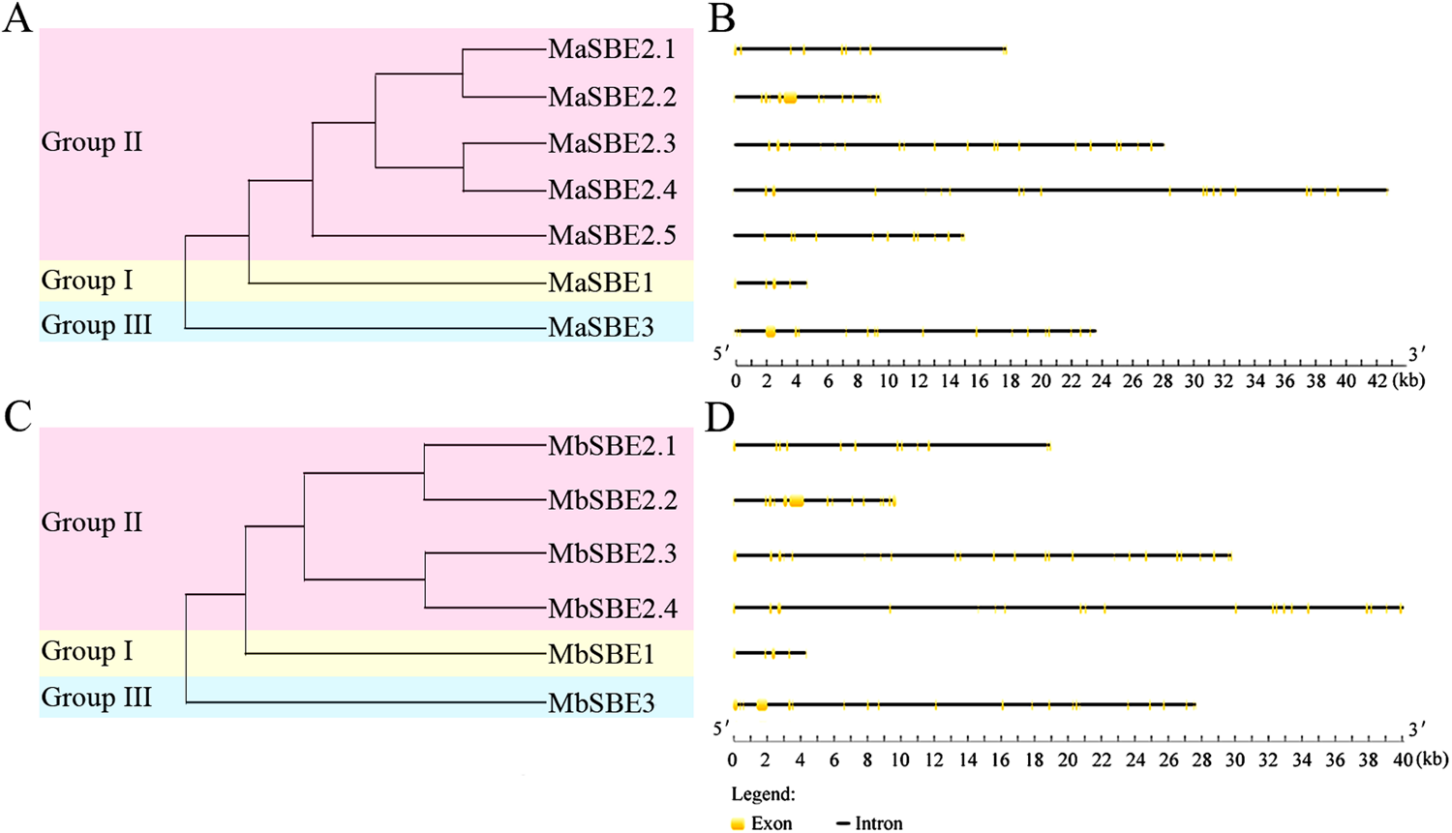
Gene structure analyses of *MaSBE* and *MbSBE* family genes. **a** Phylogenetic tree of *MaSBE* genes from the banana A-genome. **b** Exon–intron structure analysis of *MaSBE* genes. **c** Phylogenetic tree of *MbSBE* genes from the banana B-genome. **d** Exon–intron structure analysis of *MbSBE* genes. Three subgroups of *MaSBE*s and *MbSBE*s (groups I, II, and III) were formed on the basis of phylogenetic relationships. The black lines indicate introns; the yellow boxes indicate exons

The evolutionary characteristics of the *MaSBE* and *MbSBE* family members were further verified by exon–intron structural analysis (Fig. 2a, c). The exon–intron organization of the *MaSBE* and *MbSBE* genes clearly differed in groups I (5 exons), II (10–24 exons), and III (20–22 exons) (Fig. 2b, d), implying that evolutionary and functional divergence occurred among these three groups of *SBE* genes in banana.

To further assess the protein structural divergence, the transit peptides, polypeptides, and conserved domains of *MaSBE*s and *MbSBE*s were further analyzed in silico. Transit peptides were identified in all putative MaSBE and MbSBE proteins (Fig. S4). It was predicted that there are numerous polypeptides with numbers ranging from 13 (MbSBE2.2) to 67 (MbSBE2.3) and that they also have 1,4-alpha-glucan-branching enzyme activity or alpha-amylase activity (Table S4). All three domains, including the alpha-amylase domain, C-terminal all-beta domain, and carbohydrate-binding module 48 (CBM_48), were found in four *SBE*s, SBE-1, -2.1, -2.3, and -2.4, which is consistent between the A- and B- genomes. However, 0–2 domains were found in the remaining SBE members (Tables 1 and 2).

### Spatial expression features of banana *SBE* genes

**Fig. 3:**
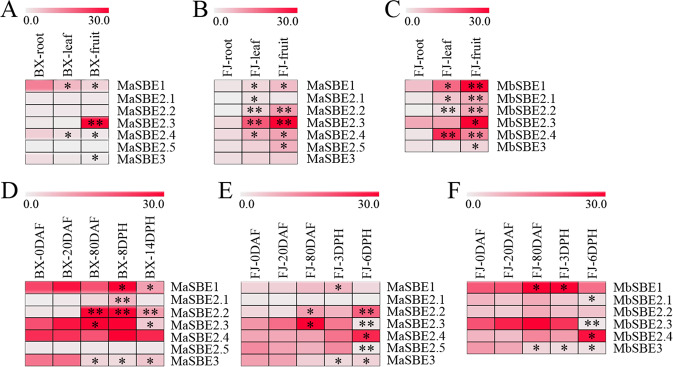
Expression of *MaSBE*s and *MbSBE*s in various tissues and at various developmental stages. **a** Expression patterns of *MaSBE*s in the roots, leaves, and fruits of the BX (AAA genotype) banana variety. **b** Expression patterns of *MaSBE*s in the roots, leaves, and fruits of the FJ (AAB genotype) banana variety. **c** Expression patterns of *MbSBE*s in the roots, leaves, and fruits of the FJ (AAB genotype) banana variety. **d** Expression patterns of *MaSBE*s throughout the processes of fruit development and ripening in the BX (AAA genotype) variety. **e** Expression patterns of *MaSBE*s throughout the processes of fruit development and ripening in the FJ (AAB genotype) banana variety. **f** Expression patterns of *MbSBE*s throughout the processes of fruit development and ripening in the FJ (AAB genotype) variety. BX BaXi Jiao (*M. acuminata*, AAA, cultivar Cavendish), FJ Fen Jiao (*M. acuminata*, AAB, cultivar Fenjiao), DAF days after inflorescence emergence from the pseudostem, DPH days post-harvest. The heat map was generated using the RPKM values of the *MaSBE*s and *MbSBE*s. The color of the red–white scale shows the differences in gene expression changes. The asterisks indicate significant differences compared to BX-roots, FJ-roots, BX-0DAF, or FJ-0DAF (*p < 0.05; **p < 0.01)

*SBE* expression in different banana plant tissues was investigated by comparative transcriptome analyses using RNAs derived from leaves, roots, and fruits of BX (AAA) and FJ (AAB). In BX, six *MaSBE* genes, *MaSBE-1*, *-2.1*, *-2.2*, *-2.3*, *-2.4*, and *-3*, were expressed in all the tested tissues of BX, whereas the expression of *MaSBE-2.5* was not discernible (Fig. 3a, Table S7). In FJ, seven *MaSBE*s (*MaSBE-1*, *-2.1*, *-2.2*, *-2.3*, *-2.4*, *-2.5*, and *-3*) and six *MbSBE*s (*MbSBE-1*, *-2.1*, *-2.2*, *-2.3*, *-2.4*, and *-3*) were expressed in all the tested tissues (Fig. 3b, c, Table S7); the expression of *MaSBE-2.3* and *MbSBE-1*, *-2.3*, and *-2.4* was significantly greater than that of other members. Among the highly expressed genes, *MaSBE2.3* and *MbSBE2.3* presented not only the highest expression level (RPKM > 33) but also a high level of tissue specificity in the fruits of both genotypes. The consistently high expression in the fruits of both genotypes may imply that *SBE2.3* is a key player in banana fruit development and starch accumulation.

### Temporal expression pattern of banana *SBE* genes

The temporal expression pattern of banana *SBE*s was analyzed by using developing banana fruits and harvested banana fruits under two different storage durations (Fig. 3d–f, Table S8). In BX, six *MaSBE* genes, *MaSBE-1*, *-2.1*, *-2.2*, *-2.3*, *-2.4*, and *-3*, were expressed during the processes of fruit development and ripening (Fig. 3d). In FJ, six *MaSBE*s (*MaSBE-1*, *-2.2*, *-2.3*, *-2.4*, *-2.5*, and *-3*) and six *MbSBE*s (*MbSBE-1*, *-2.1*, *-2.2*, *-2.3*, *-2.4*, and *-3*) exhibited various expression changes during fruit development and ripening, among which *MaSBE-1* and *-2.3* or *MbSBE-1* and *-2.3* showed significant upregulation during fruit development from 0 to 80 DAF and downregulation during the ripening process. In both genotypes, *MaSBE2.3* and *MbSBE2.3* maintained the highest expression levels throughout fruit development (RPKM > 11). This genotype-based temporal expression pattern warrants further functional characterization of *SBE2.3* in banana.

### Differential expression analysis of banana *SBE* genes via qRT-PCR

**Fig. 4:**
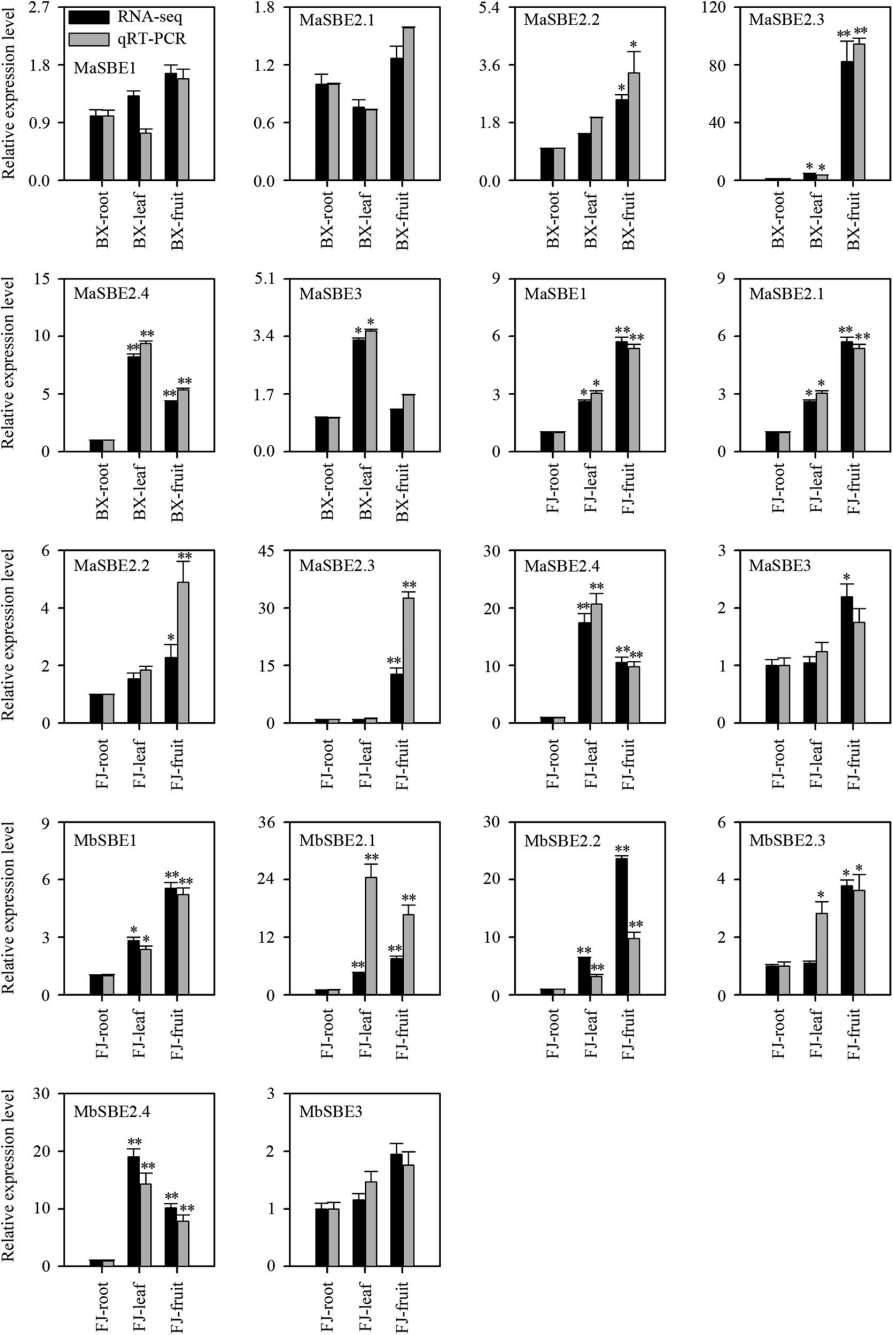
Relative expression of *MaSBE*s and *MbSBE*s in various tissues from BX and FJ banana fruits via qRT-PCR. BX BaXi Jiao (*M. acuminata*, AAA, cultivar Cavendish), FJ Fen Jiao (*M. acuminata*, AAB, cultivar Fenjiao). Biological replicates were tested in triplicate, and the asterisks indicate significant differences compared to BX-root or FJ-root (*p < 0.05; **p < 0.01)

**Fig. 5:**
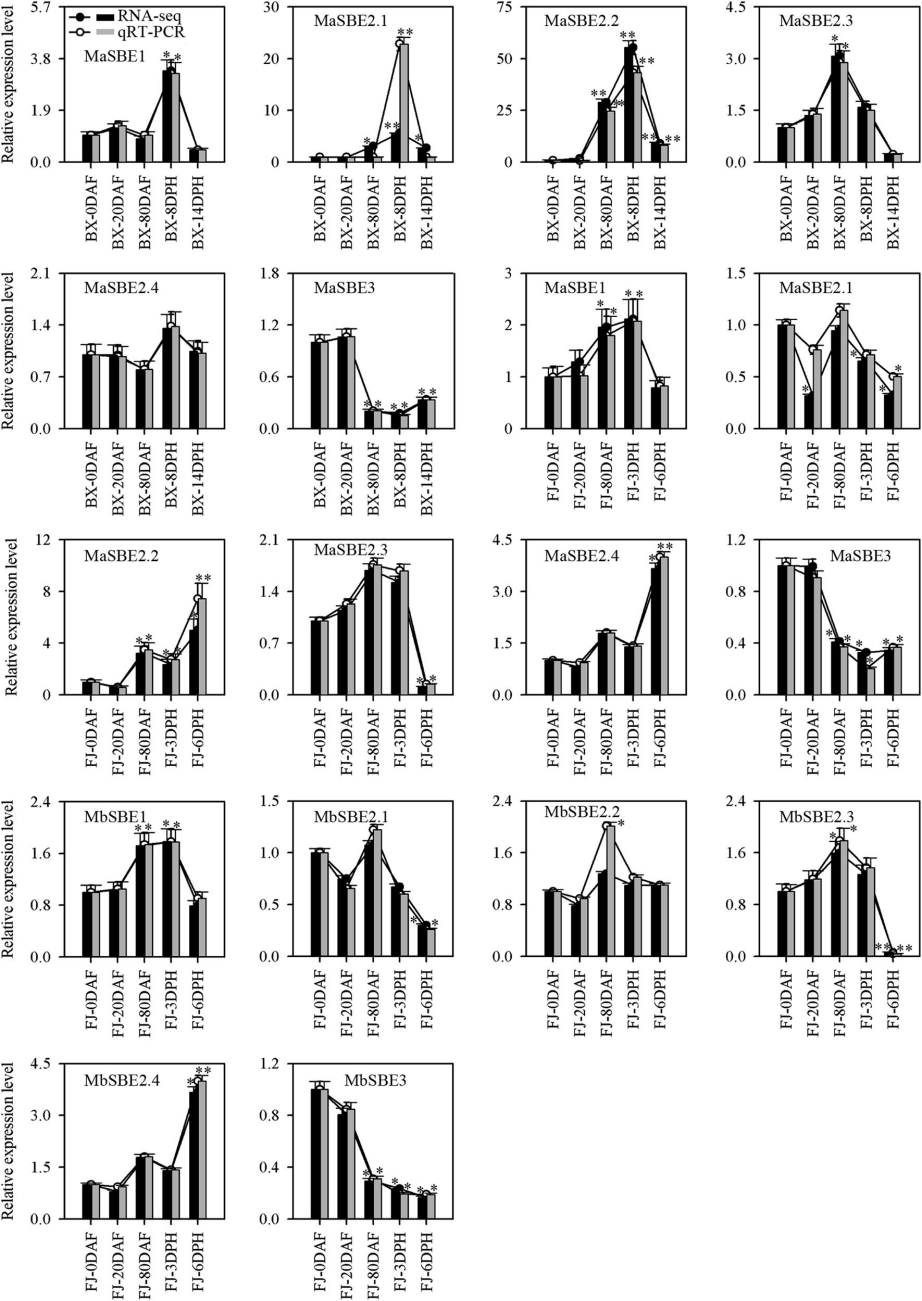
Relative expression of *MaSBE*s and *MbSBE*s at various developmental stages from BX and FJ banana fruits according to qRT-PCR. BX BaXi Jiao (*M. acuminata*, AAA, cultivar Cavendish), FJ Fen Jiao (*M. acuminata*, AAB, cultivar Fenjiao), DAF days after inflorescence emergence from the pseudostem, DPH days post-harvest. Biological replicates were tested in triplicate, and the asterisks indicate significant differences compared to BX-0DAF or FJ-0DAF (*p < 0.05; **p < 0.01)

The RNA-seq results showed that six *MaSBE*s (*MaSBE-1*, *-2.1*, *-2.2*, *-2.3*, *-2.4*, and *-3*) and six *MbSBE*s (*MbSBE-1*, *-2.1*, *-2.2*, *-2.3*, *-2.4*, and *-3*) exhibited variable expression in the different tissues or at various fruit developmental stages. This transcriptomic expression profile was verified by qRT-PCR (Figs. 4 and 5). Following normalization, the expression level of each gene of BX-root, FJ-root, BX-0DAF or FJ-0DAF was considered “1,” and the relative expression level of each gene in other tissues or at other development and ripening stages was calculated by comparing the expression to that of BX-root, FJ-root, BX-0DAF, or FJ-0DAF. All of the tested banana *SBE*s, except *MaSBE3* and *MbSBE3* in the FJ fruit (Fig. 4) and *MaSBE2.1* in the BX fruit at 8 DPH (Fig. 5), exhibited consistent expression patterns according to the RNA-seq expression analysis. Moreover, the correlation coefficients for both RNA-seq and qRT-PCR in the different tissues and at different developmental stages were greater than 0.8783 and 0.8386, respectively (Table S9), implying that the RNA-seq analysis in this experiment could provide a suitable expression assessment for both banana genotypes.

### MaSBE2.3 is essential for fruit amylopectin biosynthesis in banana fruit discs based on transient VIGS and overexpression analyses

**Fig. 6:**
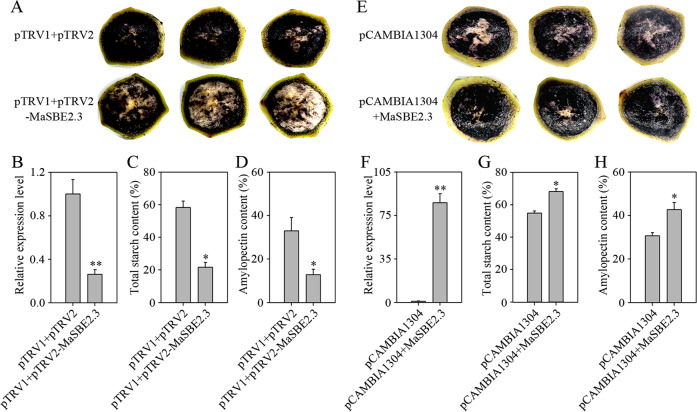
Transient downregulation and overexpression of the *MaSBE2.3* gene in banana fruit discs (BX, 80 DAF). **a** I_2_-KI staining of *MaSBE2.3-*downregulated banana fruit discs. **b** Expression level of the *MaSBE2.3* gene in *MaSBE2.3-*downregulated banana fruit discs. **c** Change in total starch content in *MaSBE2.3-*downregulated banana fruit discs. **d** Change in amylopectin content in *MaSBE2.3-*downregulated banana fruit discs. **e** I_2_-KI staining of *MaSBE2.3-*overexpressing banana fruit discs. **f** Expression level of the *MaSBE2.3* gene in *MaSBE2.3-*overexpressing banana fruit discs. **g** Change in total starch content in *MaSBE2.3-*overexpressing banana fruit discs. **h** Change in amylopectin content in *MaSBE2.3-*overexpressing banana fruit discs. Biological replicates were tested in triplicate, and the asterisks indicate significant differences compared to pTRV1+pTRV2 (empty vector control) or pCAMBIA1304 (empty vector control) (*p < 0.05; **p < 0.01)

Based on the temporal and spatial expression patterns, *MaSBE2.3* showed not only significantly higher expression in the developing banana fruits than in the somatic tissues (Fig. 3a) but also a trend of increasing expression during fruit development (Fig. 3d). To further investigate the function of *MaSBE2.3* in amylopectin biosynthesis, the expression of *MaSBE2.3* was modulated through VIGS in BX fruit discs (80 DAF). As illustrated in Fig. S5, following 3 days of incubation on MS media, relative to the fruit discs transformed with empty vector controls, *MaSBE2.3-*VIGS banana fruit discs showed lighter colors of I_2_-KI staining (Fig. 6a). Similarly, *MaSBE2.3* expression, total starch, and amylopectin contents decreased in the VIGS fruit discs, as evidenced by the respective qRT-PCR analysis and total starch and amylopectin content determination compared with those of the control fruit discs (Fig. 6b–d). On the other hand, the fruit discs transiently overexpressing *MaSBE2.3* appeared to be darker following I_2_-KI staining compared with those expressing the empty vector control (Fig. 6e). Further quantitative analysis revealed that the expression level of *MaSBE2.3* and the total starch and amylopectin contents increased by approximately 80-, 1.3- and 1.4-fold, respectively, in the *MaSBE2.3*-infiltrated BX fruit discs (80 DAF) compared with the control fruit discs (Fig. 6f–h).

### Activation of the *MaSBE2.3* promoter by MaARF2/12/24 or MaMYB3/308 in yeast

**Fig. 7:**
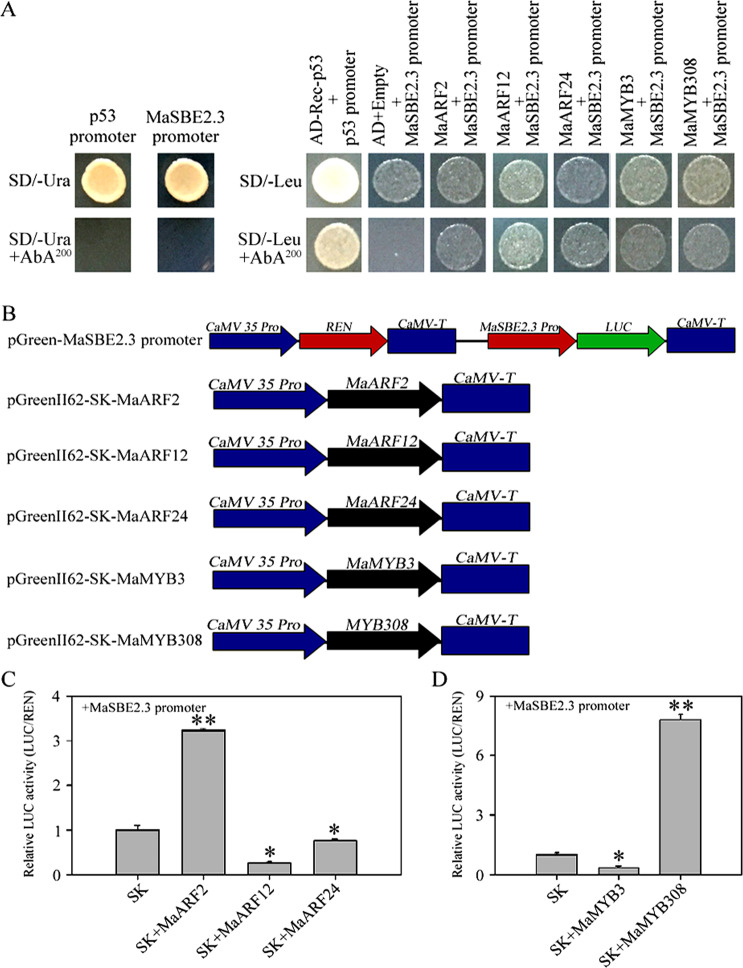
Activation of the *MaSBE2.3* promoter by MaARFs and MaMYBs in yeast or in a transient expression system. **a** Physical interactions between MaARFs, MaMYBs, and the *MaSBE2.3* promoter according to yeast one-hybrid analysis. The autoactivation of the promoters was determined on SD/-Ura+AbA media, while the interaction between TFs and the promoter was tested on SD/-Leu+AbA media. **b** Schematics of transient expression vectors. **c**, **d** LUC activity resulting from the transient coexpression of the *MaSBE2.3* promoter and *MaARF*s or *MaMYB*s in *Agrobacterium tumefaciens* strains. There were three replicates. The data are presented as the means ± SDs. The statistical significance of the differences was assessed by ANOVA (*p < 0.05; **p < 0.01)

In the Y1H-based library screening when the *MaSBE2.3* promoter was used as bait, 30 colonies were obtained and characterized by plasmid DNA sequencing (Table S10). Among them, there were three MaARFs and two MaMYBs. In subsequent verification using individual Y1H assays, the yeast clones harboring pAbAi-MaSBE2.3+pGADT7-MaARF2, pAbAi-MaSBE2.3+pGADT7-MaARF12, pAbAi-MaSBE2.3+pGADT7-MaARF24, pAbAi-MaSBE2.3+pGADT7-MaMYB3, and pAbAi-MaSBE2.3+pGADT7-MaMYB308 grew normally on SD/-Leu+AbA^200^ selective media, which is consistent with the library screening results (Fig. 7a). In the *MaSBE2.3* promoter region, a TGA element (AACGAC) compatible with ARFs and a MYB-binding site (TAACCA) compatible with MYBs were identified (Fig. S2), providing evidence for the potential molecular interactions between the *MaSBE2.3* promoter and either MaARF2/12/24 or MaMYB3/308.

### Activation of the *MaSBE2.3* promoter by MaARF2/12/24 or MaMYB3/308 in banana fruit discs

Given the interaction between the *MaSBE2.3* promoter and either MaARF2/12/24 or MaMYB3/308, as revealed by the Y1H approach, we further investigated whether MaARF2/12/24 or MaMYB3/308 directly participate in the regulation of *MaSBE2.3* expression in BX banana fruits. For this purpose, MaARF2, MaARF12, MaARF24, MaMYB3, and MaMYB308 were transiently overexpressed in banana fruit discs by *Agrobacterium*-mediated transformation using the LUC reporter system (Fig. 7b). The LUC activity was elevated by the binding of the *MaSBE2.3* promoters by MaARF2 (Fig. 7c) or MaMYB308 (Fig. 7d). In contrast, the LUC activity was significantly reduced by the interaction of the *MaSBE2.3* promoters with either MaARF12/24 or MaMYB3 (Fig. 7c, d).

## Discussion

Despite the social and economic importance of banana for its production of fresh starchy fruit, there have been relatively few studies on banana than on other high-starch crop species, such as cereals, particularly in terms of amylopectin biosynthesis and fruit development processes^6^. As a vital enzyme catalyzing amylopectin biosynthesis, SBE has also been found to play a role in seed germination, seedling growth, grain/fruit development, and maturation in many crops species^14,19–22,40,41^. In this study, through genome-wide identification, we obtained seven and six *SBE*s from *M. acuminata* and *M. balbisiana*, respectively. The putative *MaSBE*s and *MbSBE*s could be divided into three distinct subgroups according to phylogenetic relationships. The banana SBE classification is consistent with that of *A. thaliana*, *Populus trichocarpa*, and *O. sativa*^12^. Moreover, these banana *SBE*s in groups I and III were closely evolutionarily related to their orthologs in *A. thaliana* (AtSBEs) and *O. sativa* (OsSBEs), respectively. In group II, the closest orthologs of the banana *SBE*s were those from *T. aestivum*, *O. sativa*, and *Z. mays* (Fig. 1b). It appears that *SBE*s in group II had undergone numerous duplications in both the A- and B-genomes in banana, as there were 4–5 members, which is in sharp contrast to other plant species that have only one or two *SBE*s.

The phylogenetic and protein structural features of *MaSBE*s and *MbSBE*s were further characterized by their gene exon–intron organization and conserved domain structure and via polypeptide analysis. The banana *SBE*s could be classified into three distinct subfamilies according to the exon–intron organization of their structural genes (Fig. 2a, c), which is consistent with a previous report in *Malus* × *domestica*^12^. Alpha-amylase domains, C-terminal all-beta domains, and CBMs were also identified in four *MaSBE*s (MaSBE-1, -2.1, -2.3, and -2.4) and four *MbSBE*s (MbSBE-1, -2.1, -2.3, and -2.4) (Tables 1 and 2), which are the hallmarks of SBE proteins^15,16^. This finding is in agreement with findings in previous reports in other plant species, such as millet^15^ and pea^16^. However, some other members, including MaSBE-2.2 and -2.5 and MbSBE-2.2, lack one or more such core domains; consequently, the molecular mass of these members is <58.0 kDa (Table S3). In the literature, the size of active SBE enzymes was reported to be as small as 51/50 kDa in barley (*Hordeum vulgare*) caryopses^42^. Whether low-molecular-mass *MaSBE*s and *MbSBE*s are encoded by pseudogenes or have diverse functionalities distinct from classic SBEs remains unknown.

The findings that *SBE*s may play a crucial role in the fruit development process have been reported in several plant species, such as *M*. × *domestica*, *P*. *vulgaris*, and *C*. *mollissima*^12,20,22^. The *M*. × *domestica SBE1* was reported to be expressed at a high level in developing fruits, suggesting that it plays regulatory role in fruit development and amylopectin accumulation^12^. It has also been found that high levels of *SBEI* and *SBEII* expression are important to seed development in *C*. *mollissima*^20^ and *P*. *vulgaris*^22^. In the current study, among the seven *MaSBE*s and six *MbSBE*s, *SBE2.3* was identified as the only gene whose expression was upregulated during fruit development and downregulated during fruit ripening in the BX and FJ genotypes (Fig. 3d–f), implying that *SBE2.3* may be a pivotal player in banana fruit development. Moreover, the expression of other *SBE* genes varied greatly between these two genotypes. For example, the expression level of *MaSBE1* at 0, 20, and 80 DAF was 7-, 9-, and 3-fold higher in BX than in FJ. Similarly, *MaSBE2.4* showed a significantly higher level of expression in BX than in FJ throughout the entire fruit development process. Compared with FJ (AAB genotype), BX (AAA genotype) is a banana variety known to produce higher yields, and the quality of its fruits is better, with a longer shelf-life^31^. This finding is in agreement with findings of previous reports suggesting that, in banana, the A-genome contains relatively more functional genes that are associated with fruit quality and yield traits than the B-genome contains; thus the A-genome could be used as a valuable source for potential target genes in banana breeding programs^6,31^.

It has been established that *SBE*s are key players in starch biosynthesis^13^. In pea mutants, the loss of SBEII resulted in a wrinkled phenotype and 50% reduction in starch biosynthesis in the seed compared with those of wild type^43^. In maize, the loss of SBEIIb caused a 20% reduction in starch content and alterations to the morphology of the starch granules^44^. The production of high-amylose wheat starch, which comprises >70% amylose, was associated with *SBEII* suppression^45^. The simultaneous loss of both *SBEII* genes resulted in the failure of starch synthesis in *A. thaliana*^17^. In the present study, we demonstrated that the genetic downregulation of *MaSBE2.3* expression resulted either directly or indirectly in the increased turnover of total starch and amylopectin, as revealed by the reduced I_2_-KI staining (Fig. 6a–d). Such results are generally in agreement with those of reports in pea^43^, maize^44^, and *A. thaliana*^46^. In addition, consistent with previous studies in potato^1^ and chestnut^20^, the transient overexpression of *MaSBE2.3* in BX banana fruit discs at 80 DAF resulted in significant increases in the contents of total starch and amylopectin (Fig. 6g, h). However, it has been previously suggested that SBE might not be a rate-limiting enzyme in amylopectin biosynthesis, as observed in some stable transformation experiments^13^. Further, the unusually high level of *MaSBE2.3* observed in the transient expression study may not be able to reflect in planta scenarios, which warrants further exploration in future studies.

As an auxin signaling regulator, ARF affects almost every aspect of plant development, including lateral root development, embryogenesis, flower development, and seed setting^46,47^. However, it has never been implicated in starch biosynthesis. In the present study, three members of the banana ARF family, MaARF2/12/24, were found to bind the promoter of *MaSBE2.3* in Y1H experiments (Fig. 7a). For the first time, we revealed that MaARF2 activates and upregulates *MaSBE2.3* expression in banana fruit, while MaARF12 and MaARF24 suppress the activity of the MaSBE2.3 promoter in banana fruits (Fig. 7c). MYBs compose a large family of transcriptional regulators that have been shown to be involved in starch biosynthesis in hull-less barley^48^ and maize^49^. In hull-less barley, the coexpression of *MYB* and *SBE2b* was found to significantly contribute to starch biosynthesis during grain development^48^. Furthermore, maize ZmMYB14 could bind directly to the *ZmSBE1* promoter region in yeast and promote the expression of *ZmSBE1* in maize endosperm^49^. Here we identified two MaMYB members, MaMYB3 and MaMYB308, both of which were found to bind directly to the *MaSBE2.3* promoter in yeast. We demonstrated that MaMYB308 and MaMYB3 could promote and suppress *MaSBE2.3* expression, respectively, in banana fruit discs (Fig. 7d). There is no doubt that the identification of these TFs would shed additional light on the molecular regulatory mechanism of *MaSBE2.3* expression in the amylopectin biosynthesis process in banana fruits.

## Conclusions

This report represents the first study on the *SBE* gene family in banana through a genome-wide approach based on A- and B-genome data. The spatial and temporal expression profiles of individual *SBE* members were investigated in two distinct banana genotypes, and *SBE2.3* was shown to play a vital role in amylopectin biosynthesis. Using both transient downregulation and overexpression techniques, we further demonstrated that *MaSBE2.3* is essential for amylopectin biosynthesis in banana fruit. Furthermore, the identification of two classes of TFs, MaARFs and MaMYBs, which interact with the *MaSBE2.3* promoter provides further evidence of the regulatory mechanism of *MaSBE2.3* expression in amylopectin biosynthesis in banana. This study clearly improves our understanding of the molecular mechanism underlying the functionality of SBE in banana and also provides a set of useful tools for quality improvement of banana fruit through molecular breeding and genetic engineering.

## Supplementary information

Figure S1

Figure S2

Figure S3

Figure S4

Figure S5

Table S1

Table S2

Table S3

Table S4

Table S5

Table S6

Table S7

Table S8

Table S9

Table S10
