# Supplementary material for: Molecular identification of the key starch branching enzyme-encoding gene SBE2.3 and its interacting transcription factors in banana fruits
Source: Hortic Res. 2020 Jul 1;7:101. doi: 10.1038/s41438-020-0325-1 (PMC7326998; doi:10.1038/s41438-020-0325-1)
Supplement: Supplementary file 2 — Figure S2 [file 41438_2020_325_MOESM2_ESM.doc]

**Fig. S2** Sequence of *MaSBE2.3* gene promoter

AGGTTCTATATACTTAATTACTCTCAAATTTGAATATATTACCAAATGGCTTGGGTCCACGTGGGTCTTGAGATGCCATAGACCTGTTGTGTTTTCGAAGATAATTTTTTTGATAGAAAAATTGAACAGGTAATTTTTTTTCAAAGGGGTCCCTCTCCGTCACCACTTGGCTACCAATAACATATGAGGTGTTTAACTAGCTGTAACCATTGAGCTCGAGGAATAAACTTCAGTGATAAAATTTAAGGGTTCAGAAATTCATATATTAAATGCAGTAAATACAACTTATTTGATTCCAGTTGGTGCTGATGTTGATGAAAAAAAGATTGAGCTTCTCTTGTCCCAAGTCCATGGTAATGACTTGACGGAACTTATTGCTACTGGGAGAGACAAGATTGCCTCGGTGCCTTGTGGTGGTGGCGGTGCTGTTGCAGTTGCTGCAATTGCTGGAGGTGGTGGTGGTGCTTCTGCAGCCCCTGCAGTTTCTGAACCAAAGAAAGAGGAAAAGGTAGAAGAGAAGGAAGAATCAGATGATGTAAGTTACTTTTTATGATTTAACTGCAGAGTTTCTAATGTTCAGTCCTTTTGGCTGGAAACACCTTGGCTATAGATGCAGTAATCGACTTATCTCTGGATGATACCTTTGTTTTTTTTGACAAACCTTTCCTGTCCAGCCTTATTATTTGATTGACTGATTGTGCTCTAATCGCATTTGTGCAGGACATGGGCTTTAGCTTGTTCGACTGATTGGTCTACTGTAGTCCAGCAGAAGCTGCTGTTTTTGGTGCAGTAATTAGGGAAAAATCAATCTGCATTGAAGCTTCAGCTTTGATGTTTGTTGTTTATTTTCCCAAAAAAATTCCATCATGATCTCTAATTTTGTTCCTCAACATATTGCTCGGTTATATTTCTGCTTCACTCAAGTCGACAAACTTGTTAGGAGTTTGATTGAGATTTAACTTCATTGAAGACATTTGTGTTTTGTATGCCGGGTAGGCGGTCCAAGGTAACCACATTTCTTAACGGAGATTTCATTCATGTAGTTGTGCCTCGCTTCTCTAGTTTCTGTTGATACGCAAAGCCATGTCGATAGATATCAGTGGTCCTATCTTTGTTTACGCAGTTTACGTTTTAGCATGCCTGGTGCCTAGTGATAATGATTGTTGCAGATGGAGACGAATACATCAACGTCGGACGATCATATGGTGACCACTTGTCGGATGCAGATCAATATACCACCTTACCTCACTGATTGGCAACTAGGATGGAGGGTTCAGTCACACATCGCAACTTCGATGGAGGGTCGTCAACCATCCACCAAAGTAAGAATCTCAAAGCACGAACGGACGGCCTTCAACGATTTCGGTTCACACAGATTGAGGTGCGATTGGGCCCATGGGCGATCGATGAACGGTCGAGATGAGACGAATCGACCAGCTTTTTTGTTCATGAGCTACGTGCGGTATTCGAACGGACCAAAAGGCCCCACCAAAAAAAAAAGTGTTTTTACTCTTCAATTTTTATTTATTTAATATTTGATATAATGAGAGATTTTGCATAAGAAAATATATAGAATGTGACATTAACGTATTTTCGAAAATAAATAAATAAAATGAGAAAAAAGTGAACGGCCTTTACAGTGTGGGGCAGCTGAATCTGGAGAAAGCAAAGCAAAACCGTTCATTCCTCACCCCCTGCCTTCGCCAGTTTCGCTGACGCGGCCTCGTTCCCCACCTGTATTAAACGACACGTCCGCTGGGGCCCAGTTCCCATCTCCATGCACCTCTCCGCCCCACGCGGATCACCCGCCTCAGCTCCATACGA

TAACCA: MYB-binding site; AACGAC: ARF-binding site.
