# Supplementary material for: Molecular identification of the key starch branching enzyme-encoding gene SBE2.3 and its interacting transcription factors in banana fruits
Source: Hortic Res. 2020 Jul 1;7:101. doi: 10.1038/s41438-020-0325-1 (PMC7326998; doi:10.1038/s41438-020-0325-1)
Supplement: Supplementary file 3 — Figure S3 [file 41438_2020_325_MOESM3_ESM.doc]

**Fig. S3** Sequences of *MaARF2/12/24* and *MaMYB3/308*

1. **MaARF2 (2,118 bp)**

ATGGGCATCGATTTGAACACGATAGAGGAGGAGGCGGAGGAGGAGGAGGACGAGGAGTCGGAGCAGCAGCCCGCCCACCACGTCGCTTCCGCTGTGGCGGCCGAGGAGGAAGCTTGTCGGGCCGCGTCGGTGTGCCTCGAATTGTGGCACGCGTGTGCCGGGCCCCGGATTTGGCTGCCGAAGAAGGGGAGCTTGGTCGTGTACTTGCCGCAGGGGCATCTTGAGCACCTGAGGGACGGCGGCGGCGGCAGGGGGAGAGGGGGGATCGGCGGCTACGATGTGCCGCCTCATGTCTTGTGCCGTGTGGTTGACGTCAAGCTCCATGCTGATGCGGCTACGGACGACGTCTATGCTCAGCTCTCTCTTGTCGCTGAAAACGAGGAATACGAAGCAAGATTGAAGAAGGGTGAGGTTGAACAAAATGCGGAAGAAGAAAATGATGAATCTATAAGCAAGTCGCTGATTCCCCATATGTTCTGCAAGACCCTCACTGCCTCTGACACGAGCACACATGGAGGGTTCTCTGTGCCACGCCGAGCTGCTGAGGACTGTTTCCCTCCCCTGGATTATAAACAGCAGAGGCCTTCGCAGGAGCTCATCACAAAAGATTTGCATGGCACTGAATGGAGGTTTCGACATATCTACAGAGGTCAACCGCGTAGGCATCTTCTTACAACTGGATGGAGTGCATTTGTAAATAGGAAGAAGCTCATCTCAGGGGATGCGGTGCTCTTTCTTCGGGGAAATGATGGGATGCTCAGATTGGGTGTCAGGAGAGCAGCTCAATTTAAAAACATCTGTCCAGTTTCGGAACATCAAAGTGGGAATATGAACTTAGCCGCGTTTGCTGTTGTTGCAAATGCTGTGTCCGACAAAAATGTCTTTGACATCTATTATAACCCAAGGGTGAGCTCGTCAGAGTTCATAATTCCATATCGGAAATTTGTGAAAAGTTTATCTAATTCCATTTCTGTGGGAATGAGGTTTAAACTGCTATATGAAGGTGACGATGCCACAGACAGAAGGTCCACAGGACTGATAACTGGGATCAGTGACATGGACCCTGTAAGATGGCCTGGTTCAAAGTGGAGATGCCTTTTGGTAAACTGGGATGATGTTGTAAATGCTAATCAACAAACTAGGTTATCACCATGGGAAATCAAACCAACCTGTTCAGTTTTAAGCTCTGGAAGCTTGTCGACAACAGGTTGCAAGAGGGCCAAAGTTACTCTTCCCTCAGTCAATATGGATTTCCCTATTCCAAATGGAAATCAATGTCTGGACTTGAGGGAATCTGCAAGTTTCCATAAGGTCTTGCAAGGTCAAGAATTTTCGAGGTTTAGAATTCCAAGTAGTGTTGGTGTACTAGCCTCTCATGTTTCTGAGGTTGAAAAATGTCAGCACTCAGAGGGCTTTAGCAAATCTCTCGGACTCCACAAGGTCTTTCAAGGTCAAGAAGTTTTCTCAAACCATCCACCAATCCTTGGAGCTCATTCTGATGCTGATGCAAGGAATGGTGTGTATGGCCTTTTTGATGGTCTTCATACATTTCATGCTGTAAGCAGATTGTCTACAGCATCTTTGGGATATGGCACTATTGTTCAACCATCATCTCCATCAATTCAAGCATCATCCCCATCCTCGGTGTTGATGTTTCAAGAAGCAAGTTCCAAGACATCAATGGTCCAGCCTGTGCCATGCAGGAATGGTCAAGATGGTGGTGACGGTGGCAGCTGTTTTGCCAACTTAACTGGCATGGAAGCCTTGCATAGAAAAGAAGCAACCTTGCCAATCTGGCCTCCGATTATGGGTTTTCATTTTGCCAATCAGCAACACAAAATGATTGAAGTCCATGCTCCCATCTTGGATAATAAGTTGGACACACAAAATGACCAGAATGTCAGCCGAAATGGTTGCAGACTTTTTGGCTTTTCCTTGACTGAGAAGATTCCTGTAGCAGATTCAGTTGGCAAACCTCTTCCTGTCTCTTCAACCTCAACTCAGGTCAAGCTTGATGCTGCCTTCTCGACTTCAGTGGCTCAAACACCTGCTAAGCCTGTCGGTTGCAGTTGCAATGGAATAAGTGCAGCTTACACCATGTGTACTGCTCCATTTTAG

1. **MaARF12 (2,406 bp)**

ATGAAGCTTTCCACTGTTGGTATCAGCCAGCAAGCACCGGAAGAGGAGGAGAAGAGATGTTTGGATTCGGAGCTCTGGCACGCCTGTGCCGGGCCGCTGGTTTGCTTGCCGACGGCCGGTACTCGAGTGGTCTACTTCCCTCAGGGTCACAGTGAGCAGGTCGCTGCATCGACGAACAAGGAAGTGGAGGGCCATATCCCCAATTACCCGAGCTTGCTGCCTCAGTTGCTCTGCCAGCTCCATAACGTGACGCTGCACGCAGATGTGGAAACAGATGAAGTTTATGCTCAGATGACCCTGCAGCCTCTAAGTCCCGAAGAGCAGAAAGATGCTTATTTTCCTATGGAGATGGGCATTGCGAGCAAGCAACCAACGAACTATTTCTGCAAGACTCTGACAGCGAGTGATACAAGTACCCATGGAGGTTTTTCTGTACCTCGTCGTGCAGCTGAAAAAGTCTTTCCGCCCCTGGATTTCTCTCAGCAGCCTCCAGCACAGGAGCTTATTGCTCGTGACCTTCATGATGTTGAATGGAAGTTTAGGCATATTTTCCGAGGTCAACCAAAAAGGCATCTCCTCACCACAGGCTGGAGTGTGTTCGTCAGTGCCAAGAGACTTGTTGCTGGGGATTCTGTTCTTTTTATTTGGAATGAAAAGAACCAGCTTTTGTTGGGAATAAGGCATGCTAATCGGCCACAAACTTCGACACCATCATCAGTTTTATCAAGTGACAGCATGCACATTGGACTCCTTGCTGCAGCTGCTCATGCTGCTGCTACAAATAGTCGTTTTACTATATTTTATAATCCAAGGGCCAGTCCATCAGAGTTTGTTATACCTCTTTCTAAGTATATTAAAGCAGTATTCCACGCACGTGTATCAGTTGGGATGAGATTCCGTATGCTTTTTGAGACTGAGGAATCAAGCGTTCGTAGGTATATGGGAACAATCACTGGCATAAGTGACTTGGATCCTGTTCGTTGGCCGAATTCTCATTGGAGAACTGTCCAGGTTGGTTGGGATGAATCCACAGCAGGTGAAAGACAGCGCAGAGTATCACTGTGGGAAATTGAACCTTTAACTACCTTTCCCATGTACCCTTCATTGTTCCCTATTGGCCTTAGACGTTCTTGGCATCCTGGAGCTTCCTTTCCTCATGACAATAGAGAAGAGTTTAATACTTTCATGTGGCCGAGGGGGGTTCCTGTTGATCAAGGCATGTACTCATTGAACCTCCCATCACTTGGTACGGGTCCTTGGATCCAGCAGAGGCATGAAACATTGCTGTTGGGAAGTGAGTTTGATCAGTACCAAGCCATGTGTCCTCTAGCTTTTCAGGACATACGATGTGGGGATATTCTGAAGCAGCAATTTCTACAAAATCAACAACCAATTCAATTTCTCCAACAATCCTGCACATCCAGTTCACTATTCAAGCCTCAAGACAATCAACAGCAGATTGTTAATCCCCAACCACAATGCTTAACAGAAAACCATAGACATCCGGTCCCATACCAGCAGTTGCAGCCACCGCATACTGAACAACAAAAGCAGCTGCCTCAAGAAGCAAATGTTTATACACAGGCATTTACAATGCATAACAATCATGTGCAGCGGCAATCTGCTTTGCCCTCTCCATTATTTGAAAACTCAACCATTCCTGATTCTAGTTTAAATTTCTCCTCAGTTCCCACACCAATTTCTGACCAGGATATCTTAGGATCTGCTTACCGTGAAGGAAATGTCAGTGGTTCCAACTATTCACGACTCAATCAATCCATGATAAACCATCCTGGTCAGAAATCATGGGAACTAAATTTTACGAAGTCGCAAATGATCTCTTTTGATGGTGCAGCTCTCCTTTCATCATTTCCTGCTAAGAACAGTACTGTGGGAAATGATAATTTCACAGATACCCAGACTTGCACCCTTTTTGGTTTTAGCAAAGATTCCTCCTCTCTGCTAAATAGTGCATTATCAAATTCAGGAACTGTGAATGATGTGTCAACAATGCCATACACTAGTTCGTGTTTCCAGAATTCTTTTTATGGGTATTTAGATGACTCGCCTAGCTTACTACATGGTGCAGGAGAGACTGATCTGCAATCCCAAACCTTTGTGAAGGTTTATAAGTCAGGATCGGTTGGGAGGTCTCTAGATATCTCCCGGTTCAGCAACTACGAGGAACTACGCGAGGAGCTGGGTCAGATGTTTGGTATTGAGGGTCTATTGGAGGATCCTCTTAGATCAGGCTGGCAGCTTGTATTTGTCGACAGGGAGAACGATGTGCTTCTCCTTGGAGACGACCCATGGGAGTCCTTTGTGAATAATGTTTGGTATATCAAAATACTTTCACCTGAGGATGTGCTGAAAATGGGAAAACAGGGAGTTGACTCTTTCTGTTAG

1. **MaARF24 (2,625 bp)**

ATGGCGTCGTCGGAGGTGTCGGTGAAGGGGAGTAGTCAAGGACGAGGGGATACCGTGTCCTCGGGATGTAGCGAGCTGCACGACGGCGCGCCGGTGAGGGGAAGCGACACCGAGGGGAGCCTTGGGCTTTCCGGCGGCGCGGTCGGCGGGAAAGATTCGGTGGATGCGTTGTATACGGAGCTATGGCACGCTTGCGCAGGCCCGCTGGTGACGATTCCCAGAGTGGGGGAGAGGGTCTTCTACTTCCCACAGGGTCATATGGAGCAGGTAGAAGCTTCAACAAATCAGGTAGCGGACCAGCAGATGCCTGTCTACAATCTCCCGTGGAAGATCCTCTGCCGGGTGATGAACGTCCATCTGAAGGCCGAACCAGACACCGATGAGGTATTTGCTCAGATCACTTTGCTCCCTGATTCAAAGGATGAGAACACCGTGGAGAAGGACACGCTTCAGCCTCCACCTCCACGGCCTCATGTGTATTCCTTCTGCAAGACGCTGACCGCGTCAGACACCAGCACCCACGGTGGGTTCTCAGTGCTAAGACGGCATGCTGATGAGTGCCTACCTCCGCTGGATATGAGCCAGCAGCCGCCAAGTCAGGAGCTTGTGGCGAAGGACTTGCATGGAGTTGAATGGCGCTTCCGCCACATCTTTCGTGGTCAACCGCGAAGACACCTGCTTCAAAGTGGATGGAGTGTCTTTGTTAGTTCTAAAAGGCTTGTTGCTGGGGATGCTTTTATCTTTCTAAGAGGGGACAATGGCGAACTACGTGTAGGCGTGAGACGAGCTATGAGACAGCAGACAAATGTTCCATCTTCAGTTATATCCAGTCACAGTATGCACCTGGGTGTCCTTGCTACAGCATGGCATGCTGTCAACACTGGAACCATGTTTACTGTTTACTACAAACCACGGACTTGTCCAACTGAATTCATTGTTCCTTTTGATCAATATGTTGATTCAATAAAAAACAACCATTCTATAGGAATGAGGTTCAAAATGAGATTTGAAGGTGAAGAAGCCCCAGAACAGAGGTTCACTGGCACCATTGTTGGCATTGGAGATTCTGAACCAAGCAGGTGGCCTGGATCGAAATGGAGATGCCTGAAGGTACGTTGGGATGAGGCCTCTTCAATTCCTCGTCCAGATAGGGTGTCCCCATGGAAAATTGAACCTGCTCAGTTGCCTCTGCCTCCTAACCCTATTCCTATGCCTAGGCCAAAAAGGCCTCGAACAACTGCCTTTCCTTCTTCCCCGGATTCATCTGTTCTTACAAAAGAAGCCTCTTCTAAAGTCTCTATGGACCCTTCCCAATCACATGGAGTCCCAAGGGTATTGCAAGGTCAAGACATGGCAACATTGAGAAGTACTTTTAGCAACGAGGCAGATACTGCTCAGAAGCCAATCATGTGGTTGACACATGACGAAGAGAAAAATGATGTTTCTGCTCAGAGAAGATTGGGATCTGAGAGCTGGATGCACATAAAGAGGCAGGAACCTATGTACACTGACATGTTATCAGGATTTCAGCCTTCTGGTGATTCAAGTGGGTTTCACTCACCATTTCTTGAGCAGGCCTCAGGTGATAAATTTTTTTTAAAGCCTCATTTCCGGGATCAAGAGGCCAAACATAACTGTTCTCCTGGCTTATGGTCTTTGATGCCTTCAAATTCAAATTTAAATTTGGGTGAATGCAACTTGAAAATGACTGCACATGTTGGTGAGCTATCCTTTCAGAAAGTTGGAAGTTGCATATATGGAATGCAGGGAGGCCAGTCGGAACTGAAGGGCTTAGGAGGTGATCAACAGTCATCAGATTGGTTAGGATGTTCGCTTCCCGATTCTCAAACCGATAACATGCCACAACACAGGGTCATCACACTTCAGCCCCTGGTACCGTCCCAAAATGATGTGGCAAAGTACAAAGCAAATAATAGTGGTTGCAAGCTATTTGGGTTTCATCTAAACAGCAAACCTGTGGCATCTGAATCAGTTGTACGACAGAGCAACTCTGCAGAAATTCTGATGCCACATAGCCATCTAGCTGCAGCATTGCCCCAACCGCAAGGTCTAGAGGCTGCCAAGCATTCTGAGTCATGCATAGCTGCAAAGTTGGTCTCCGCCACACTGACAAATGGTGATACAGAGAAACTTATTCAGGTTTGTCCACAGGCTTCTAAGGATGTTCAGAACAAAATGCAAGGTGGTTCAACCAGGAGTTGCACCAAGGTGCATAAGCAAGGTATTGCTCTTGGCAGATCTGTTGATCTTACCAAGTTCAATGGTTATGATGAACTGATTGCTGAGCTAGATCAAATGTTTGAATTTGAGGGTGCATTAATAGCTCCCAACAAAATTTGGCTTGTCGTATACACTGACAATGAAGGTGATATGATGCTTGTTGGAGATGATCCCTGGAATGAATTCTGTAACATGGTTCGTAAGATTTACATCTACACTCGAGAGGAGGTCCAAAGAATGAATCCAGGAACACTAAATTCTAGAGTTGAAGAGTCTCCAGCTGTTTCTGAAGAAAGGATATCTGGCAAAGAAACAAAGCGCCCTGTGCCTACTGCTAATTCTGAGAATTCTGAGGGTTGTCCTGGAGTTGATGCGGCCTTTTGA

1. **MaMYB3 (1,663 bp)**

ATGACGACAAGGTCGTGGATGGAAGTCCTTCCTCCGGCGACGGTGCCGTGTTACCCCAGCTCAAGTTGGTTCATTGGCGAGAAAATGAGTGGGGGCGGCATCGGAGGCGGAAACTGGACCCCGGAAGAGAATAAGCGATTCGAGTATGCCCTGGCGAAGTTCGACAAGGACACCCCTGACCGCTGGGAACAGGTGGCGGCGTCTATCCCCGGTAAGACCGCGTGGGACGTGGAGAGCCACTACCGGGATTTGTTGGACGATGTGAGCGACATAGAAGCCGGGCGGATCCCATGTCCTGGCTACGACTCTTCGTCTTTTACACTGGACTGGGAGACCAATTACGGCTTCGAAGCGTCCACGCAACCTTACTGCATTGGTGGGAAGAGGTCAGCAGCGCGAGCATCGGATCAAGAGAGGAAGAAAGGAGTTCCCTGGACCGAAGATGAGCACAAGCGCTTTCTGTTTGGTCTCAAGAAATATGGAAAAGGGGATTGGAGAAATATATCTCGGAATTTTGTGATCACTAGAACCCCTACCCAAGTCGCTAGTCATGCACAAAAGTACTTCATCAGACTTAATTCAGGTGGCAAAGATAAGAGGAGGTCCAGCATACATGACATTACTACTGCCAATTTGCCTGATAATAGGCCTCCTTCTCCATCTCAGTCATCCGATCCTGCCACTCAGACAAGCTTGGCTTCTACACCGCTACCGTCGGCCCCATTCTCATCGATCCTTGATTCGAGTCATCCCAATGAAGCAACTACAATTGCAACTTCTTCGGTGCAGGGGAGTACAATTCGTGCAACCAAATTATGGAGTGACACCTTATGGTCTGCAACTAGAAGATCATGCACCTCGGAGTGGCACACTCGATGCTACCGTGGTTCAGGACCATGATTTGCTATTTGAGATGCGATCGATCGAGTCATCGTCGCTGCCATGGATGATTAGTTATAGTCCAACTATCCTTTGCACTCTCAATTTCTTCTTCTTCCCAAATGAGAAGCCACTGAGAGTTACTGGTCAGCTGAATCTAGCTCGAGTTTCGATCTTTAGCTCGGGGGAGAGAGAGATGGGCAACATCTGCGGGATGATGGCGAGTTGGTTCCTGGGCGAGAAAGGGGGCGCGGGCGGCATCGGAACCGGGAACTGGACTCAAGAAGAGAACAAGCTATTCGAGCATGCCGTGGCGAAAAGGAGGTTCGACAAGGACACCCCTGACCGCTGGGAAAAGGTGGCGGCGTATATCCCCGTGAAGGCCGTGAGGGACGTCGTGAGCCATTACCGGGACTCGTTGGACTACGTGAGCGAGATAGAAGCAGGTTGGGTCCCGTGTCCTGGCCACGACTCTTCTTTGTCGATGCAGCCTCCATGCGTCGGCGGGAAAAGAGCAGCATCGGATCAAGAGAGGAAGAAAAGAGTTCCCTGGACCGACGAGGAGCACTGGCGATTTCTGCTTGGTCTCAAAAAATATGGGAAGGGGGATTGGAGAAATATTTCTCGGAATTTGGTGACCAGCAGAACCCCTACTCAGGTGGCTTCTCATGCCCAAAAGTACTTCAGCAGACTGGAAGATAAGAGGAGCTCCGGCATACATGACAGTAGTACGGCCAAATTGCTGGATAACAGGCCTCCTTCTCCATCACAGTCATCTAGCTAA

1. **MaMYB308 (726 bp)**

ATGGGGAGGTCCCCGTGCTGCGAGAAAGCGCACACCAACAAGGGGGCGTGGACCAAGGAGGAGGACGACCAGCTCATCGCCTACATCCGCGCTCACGGGGAGGGGTGCTGGCGCTCGCTGCCCAAGGCCGCCGGCCTCCTCCGCTGCGGCAAGAGCTGCCGACTCCGCTGGATCAACTACCTCCGCCCCGACCTCAAGCGCGGCAACTTCACGGAGGAGGAGGACGAGCTCATCATCAAGCTCCACAGCCTCCTCGGCAACAAGTGGTCACTGATCGCTGCGCGGCTTCCCGGGAGGACGGACAACGAGATCAAGAACTACTGGAACACCCACATCAGGAGGAAGCTGCTGAGCAGGGGAGTGGATCCCGTCACTCACCGCCCGATCAACGGCCATGGTTCCGATACAGCCACCCATTTCGAGAGGAAGCCTGCTGCGATTGGCTTCGGACCAGACCAGGAGTCGGACAAGATCAGTAGCAGCGAGGAGTCGTCGGCGTGGCAGCAGCGGCAGCCCAAGTGGCCGGACCTCAATCTGGAGCTATGTATAAGCCCTCCTTCCCAACAGCAACAACCTTTAGAGCCCGTTGATGGAGGGGGAGAGCAACCGCAAAGCCTGTGCTTTAGCTGCGGCGCTGCCGTGGGGTCGCAGAAGCGCCACAAGGAGTGCAAGTGCAGAGACCTCATTGGCCTCAGCACTGGAATGCTGGACTACAGAAACCCATAG
