# Supplementary material for: Molecular identification of the key starch branching enzyme-encoding gene SBE2.3 and its interacting transcription factors in banana fruits
Source: Hortic Res. 2020 Jul 1;7:101. doi: 10.1038/s41438-020-0325-1 (PMC7326998; doi:10.1038/s41438-020-0325-1)
Supplement: Supplementary file 4 — Figure S4 [file 41438_2020_325_MOESM4_ESM.doc]

**Fig. S4** Transit peptides of all predicted MaSBE and MbSBE proteins

1. **MaSBE1**


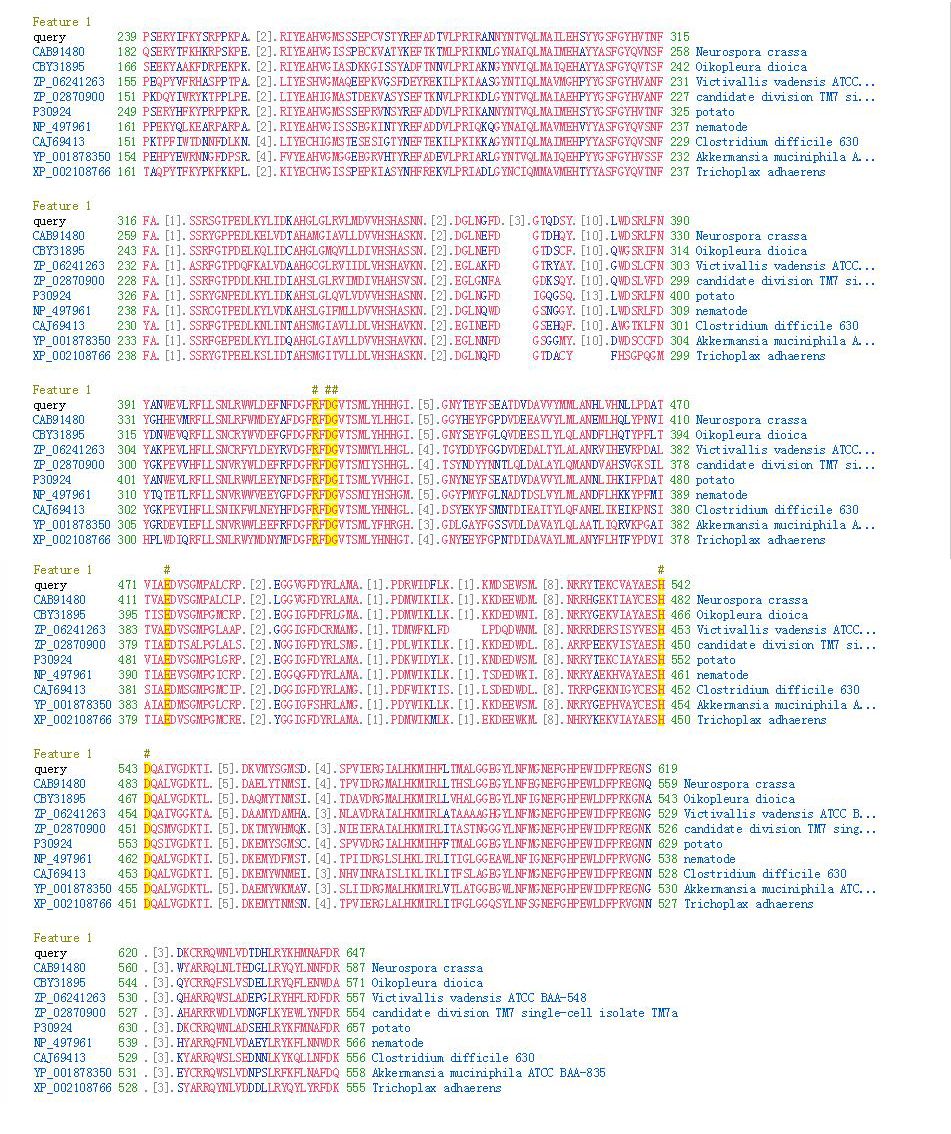


1. **MaSBE2.1**

**
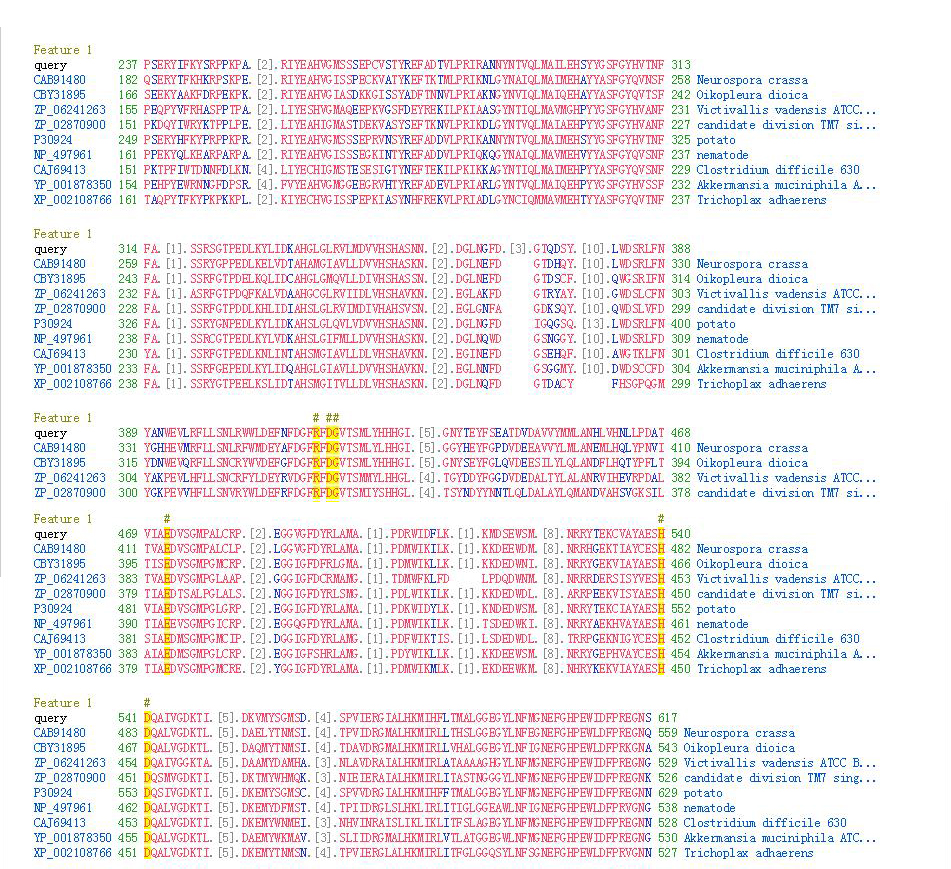
**

1. **MaSBE2.2**

**
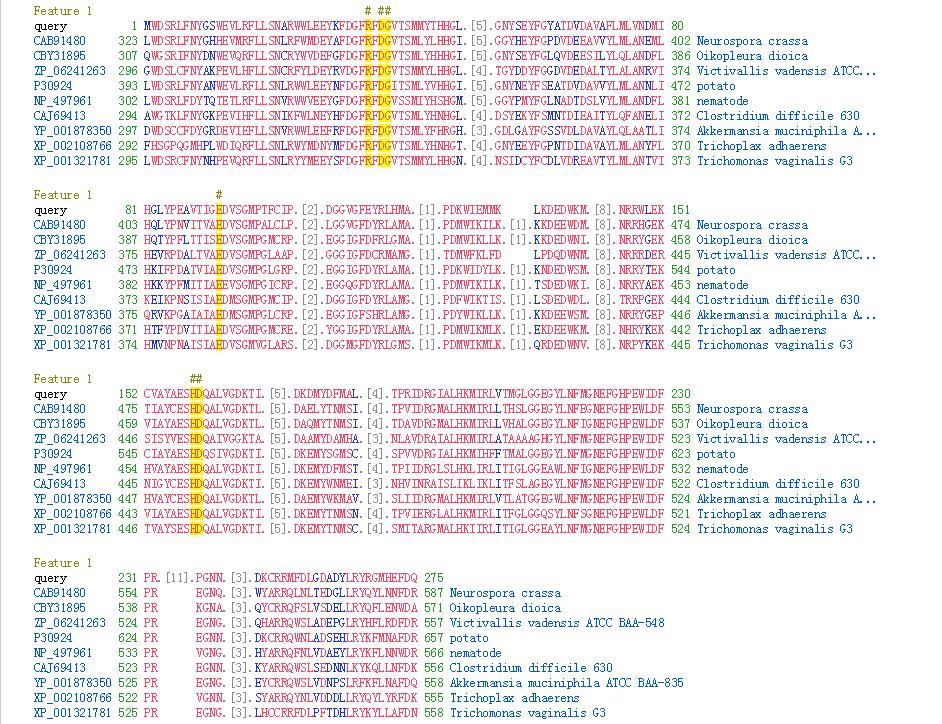
**

1. **MaSBE2.3**

**
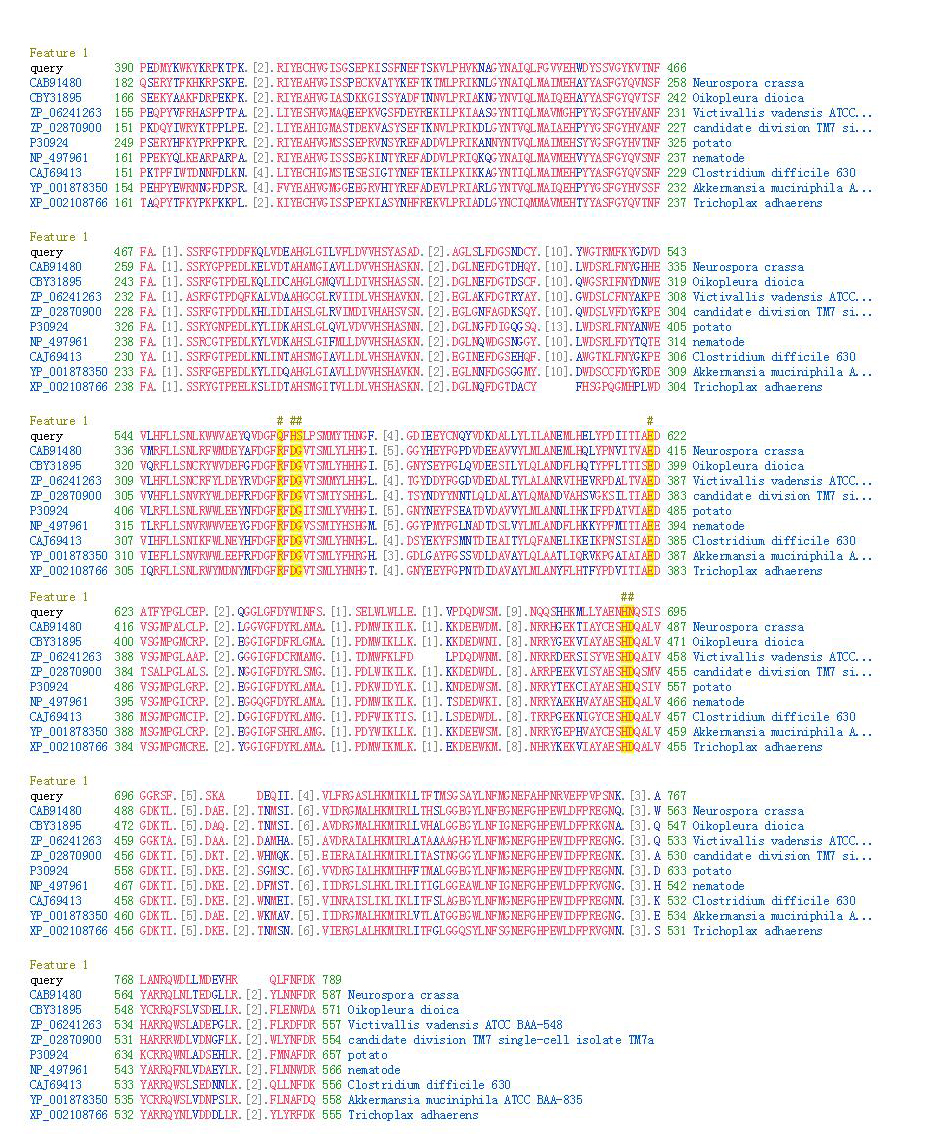
**

1. **MaSBE2.4**

**
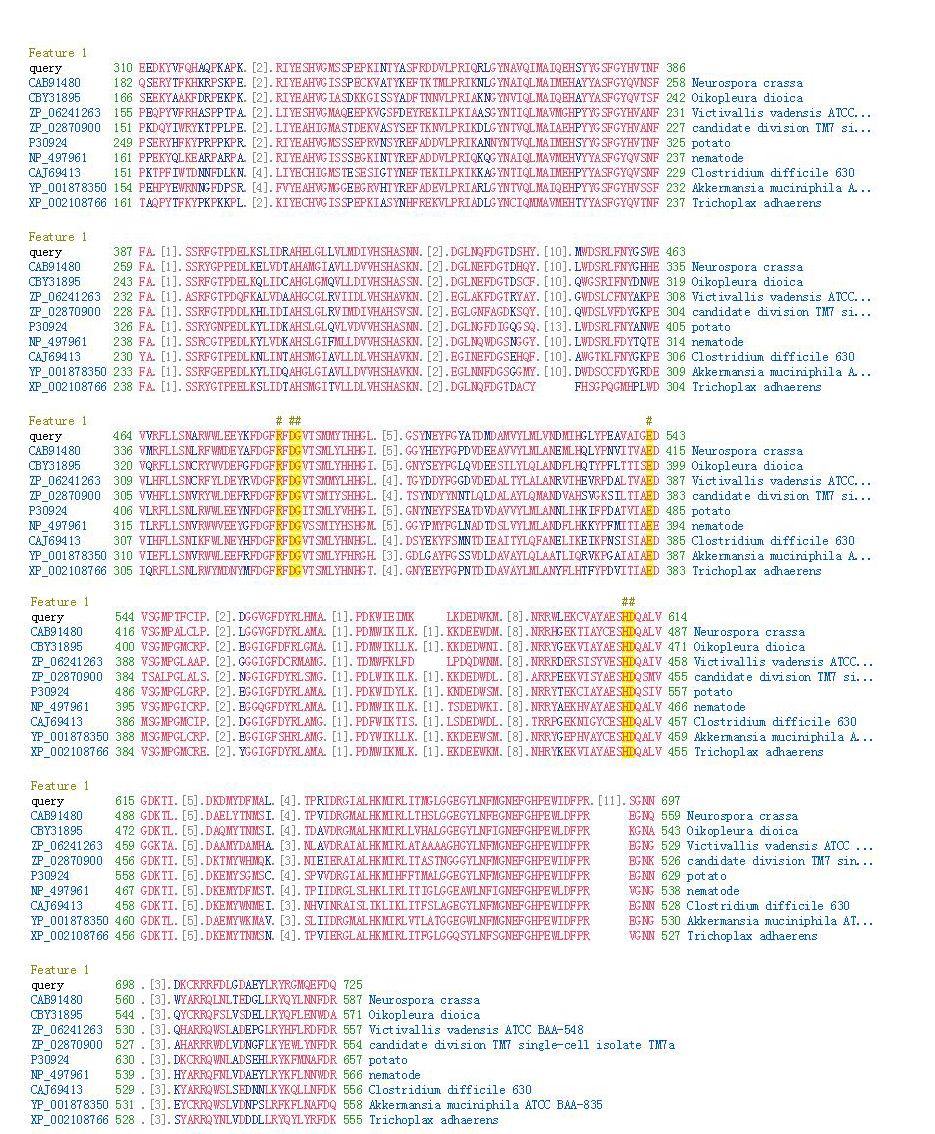
**

1. **MaSBE2.5**

**
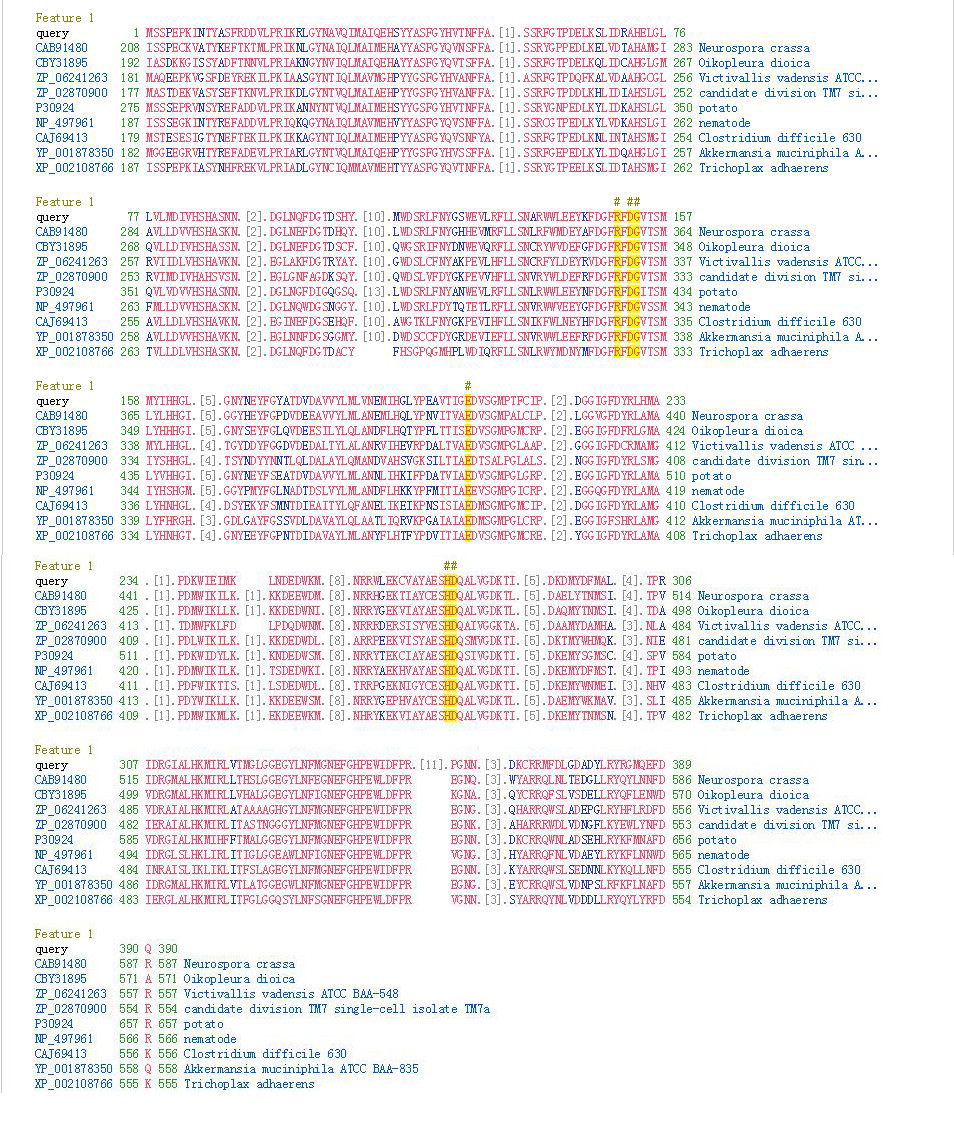
**

1. **MaSBE3**

**
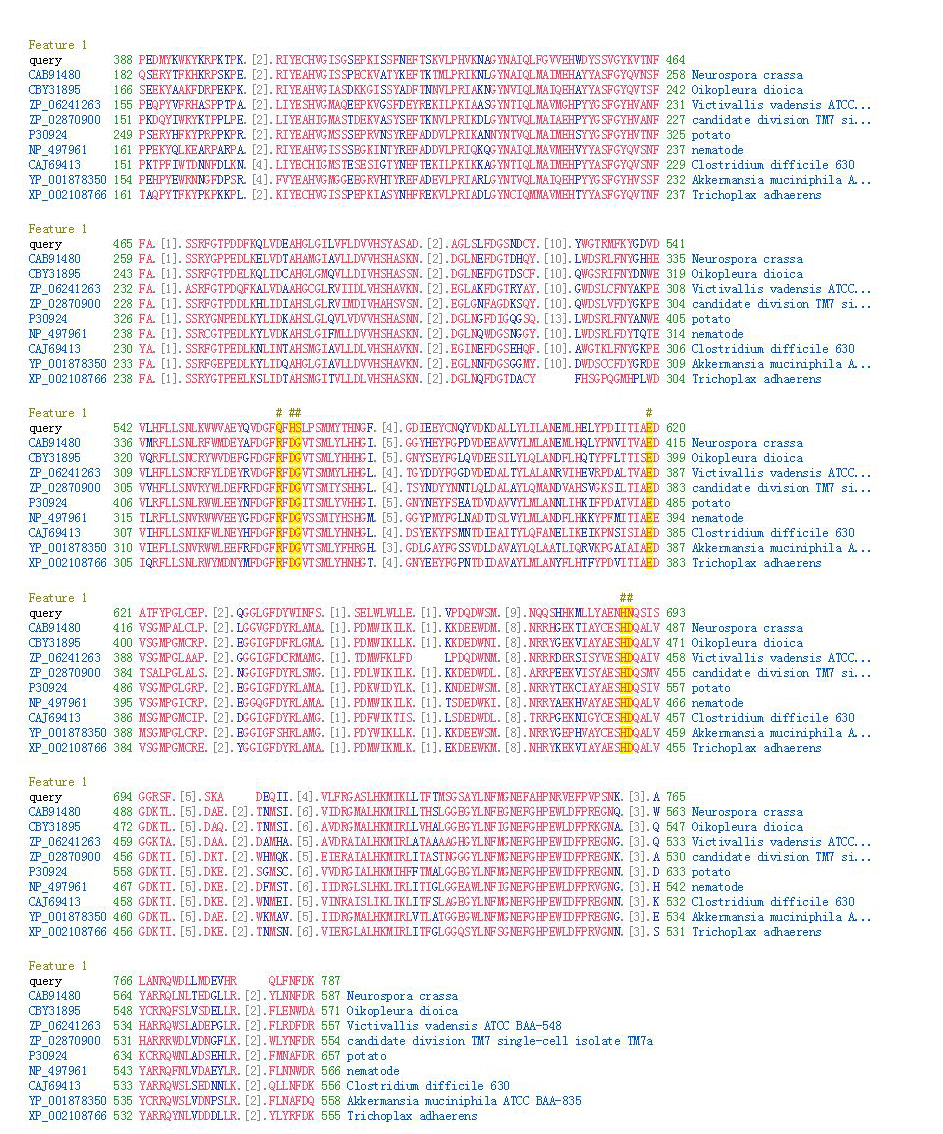
**

1. **MbSBE1**

**
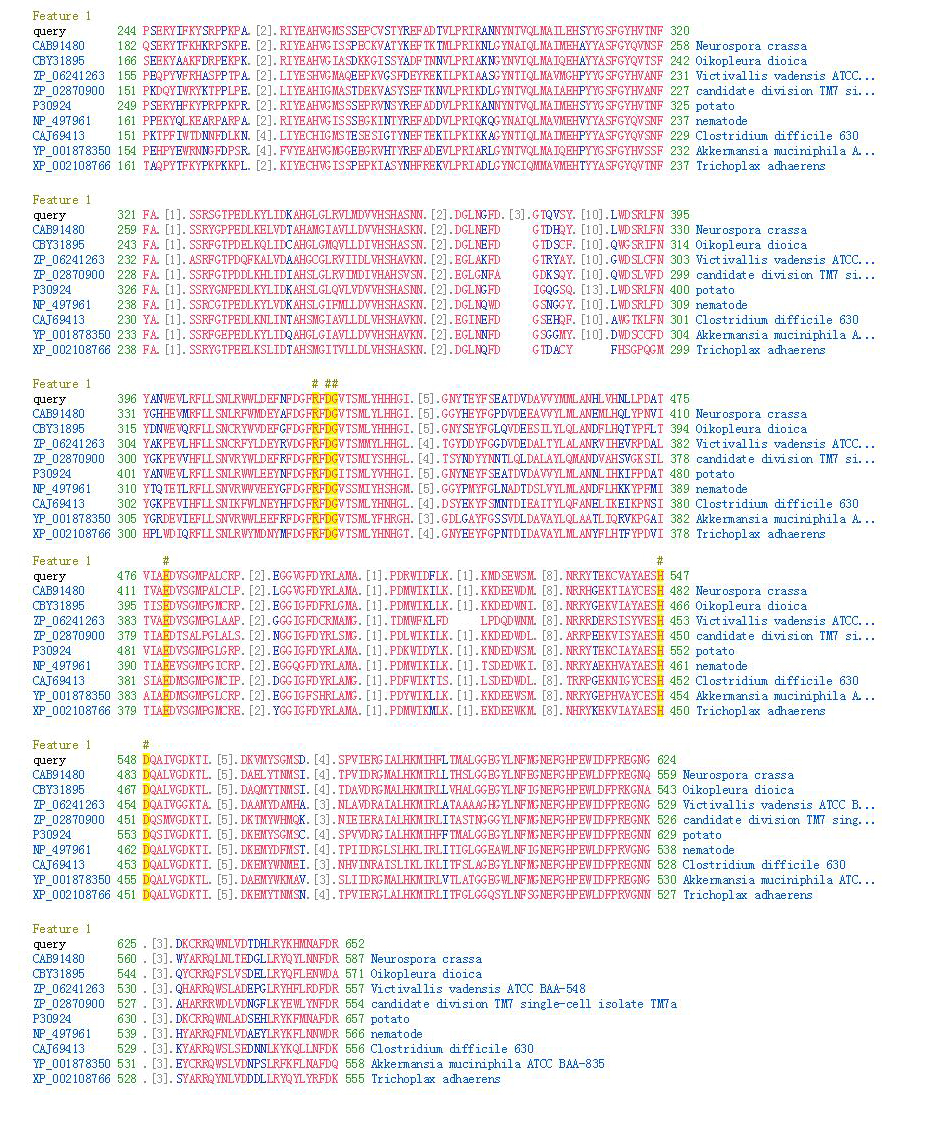
**

1. **MbSBE2.1**

**
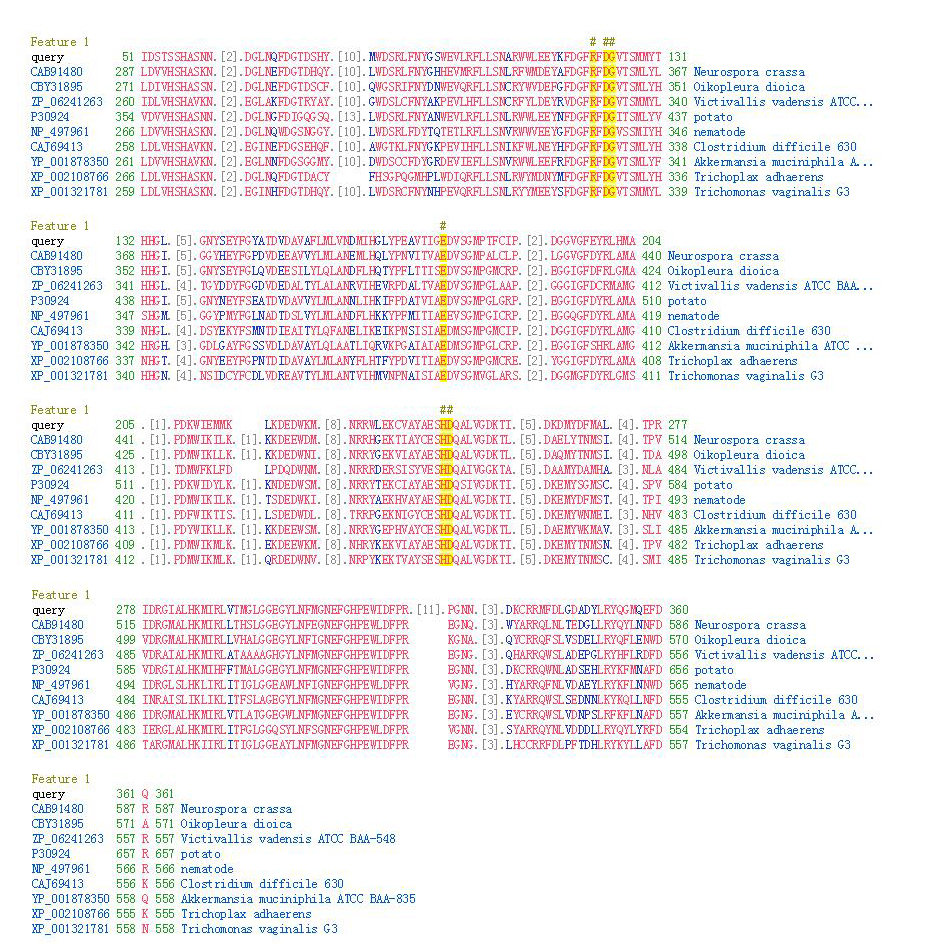
**

1. **MbSBE2.2**

**
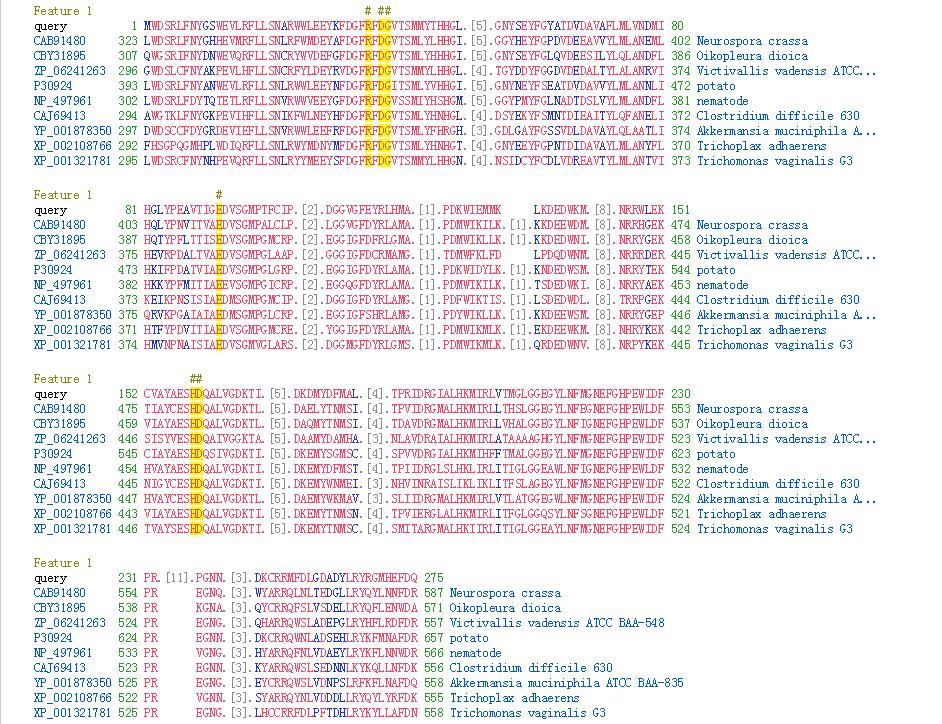
**

1. **MbSBE2.3**

**
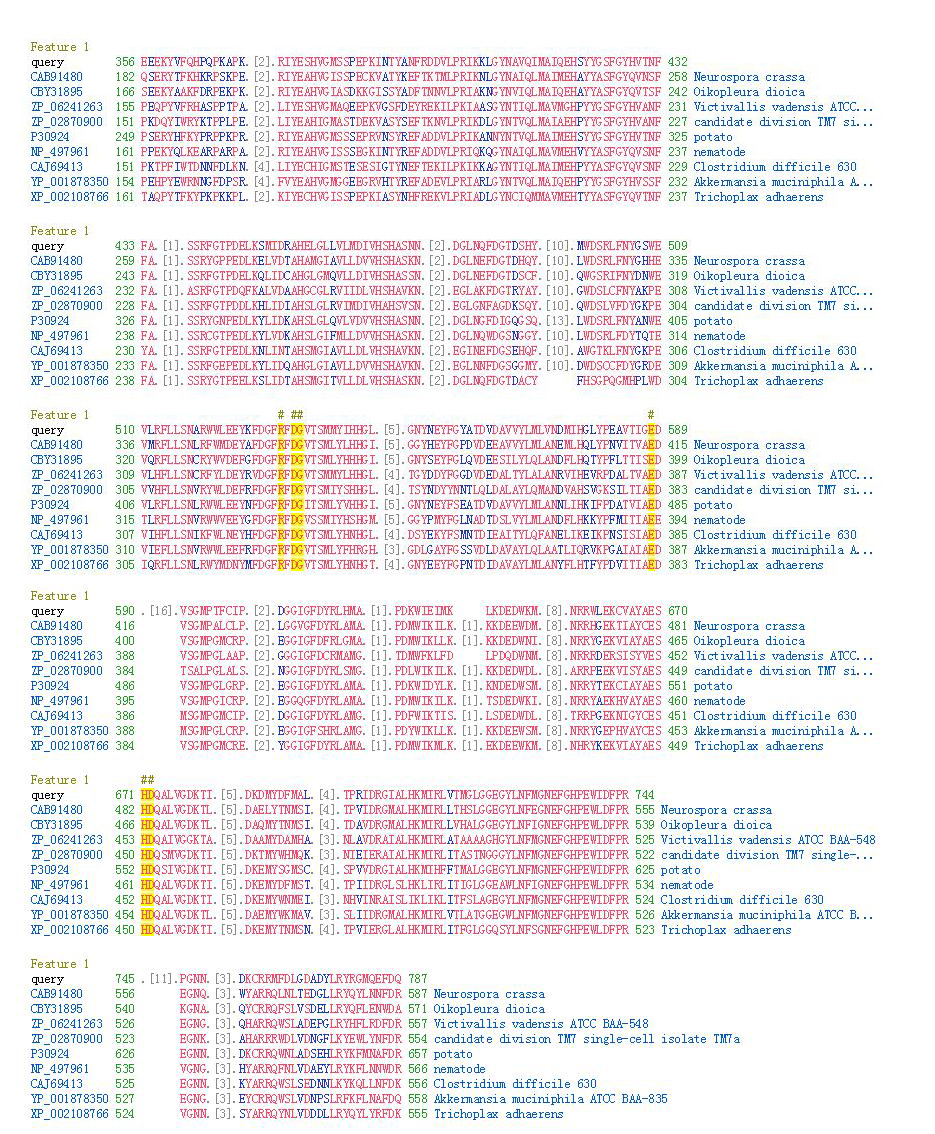
**

1. **MbSBE2.4**

**
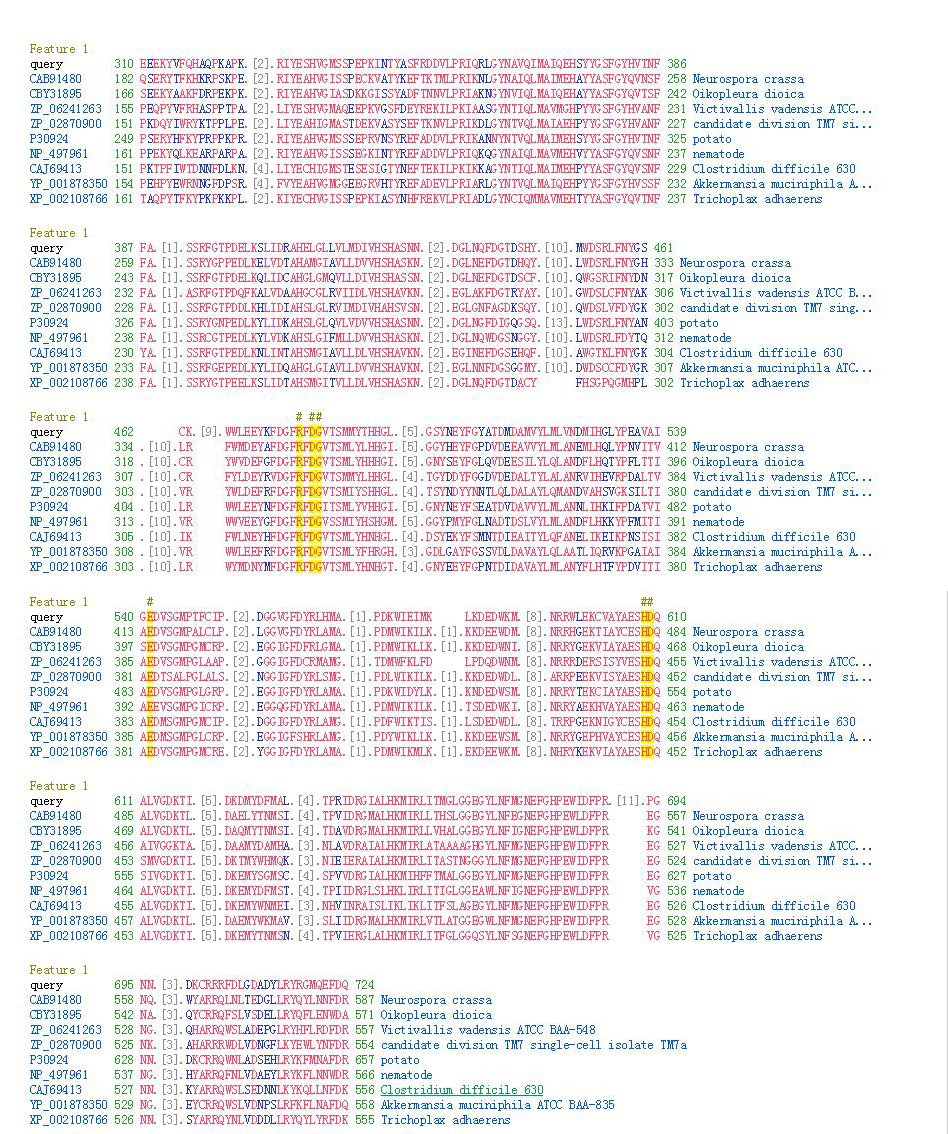
**

1. **MbSBE3**

**
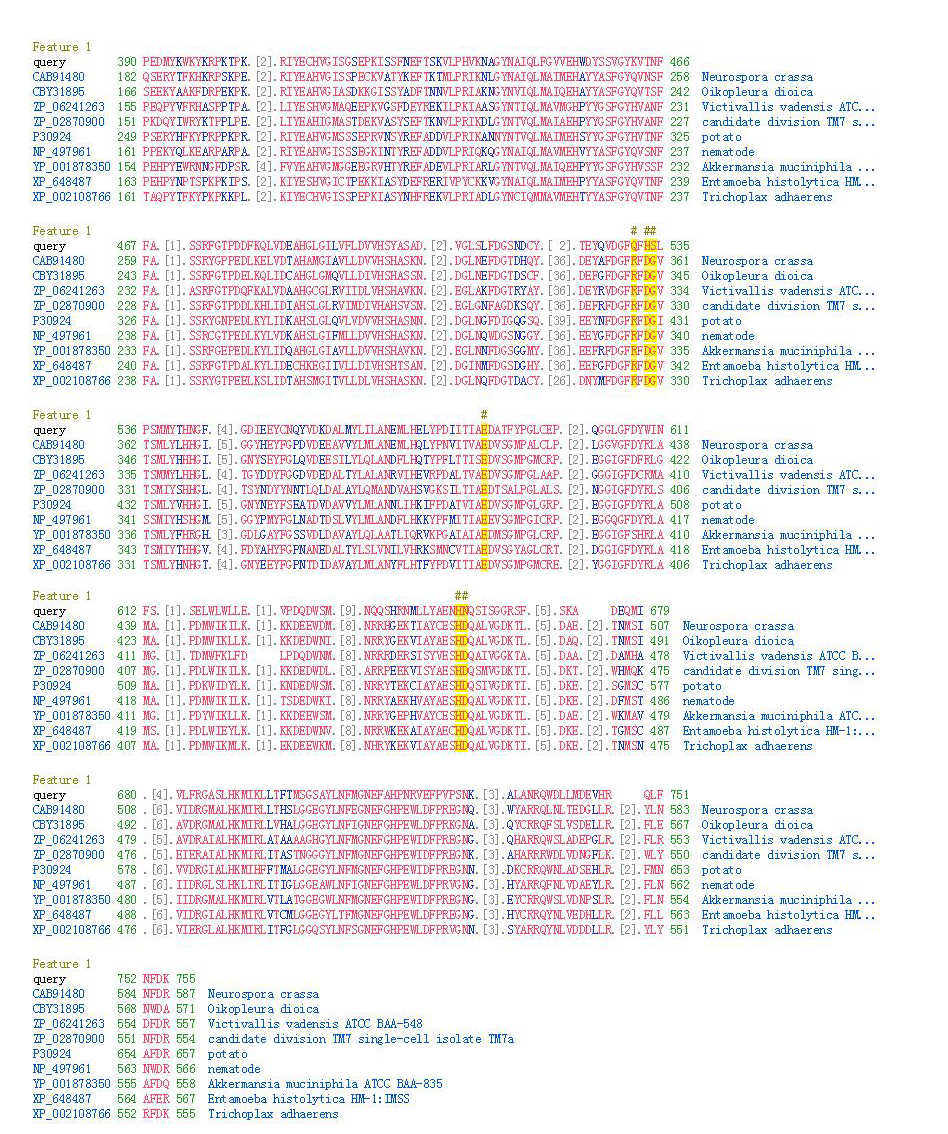
**
