# Supplementary material for: Molecular identification of the key starch branching enzyme-encoding gene SBE2.3 and its interacting transcription factors in banana fruits
Source: Hortic Res. 2020 Jul 1;7:101. doi: 10.1038/s41438-020-0325-1 (PMC7326998; doi:10.1038/s41438-020-0325-1)

**Fig.S5** I_2_-KI staining change of banana fruit disks during the incubation on MS medium after Agro-infiltration experiments


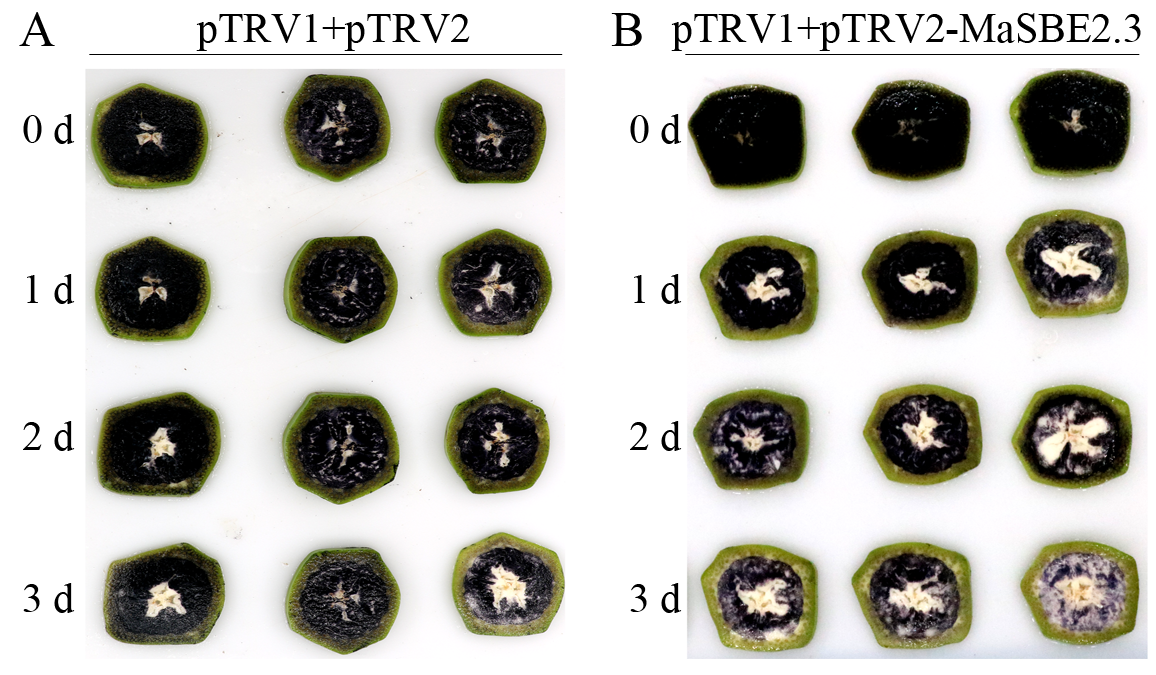

Supplement: Supplementary file 5 — Figure S5 [file 41438_2020_325_MOESM5_ESM.doc]
